# Copper(II) Oxide Spindle-like Nanomotors Decorated with Calcium Peroxide Nanoshell as a New Nanozyme with Photothermal and Chemodynamic Functions Providing ROS Self-Amplification, Glutathione Depletion, and Cu(I)/Cu(II) Recycling

**DOI:** 10.1021/acsami.4c17852

**Published:** 2024-12-25

**Authors:** Çağıl Zeynep Süngü Akdoğan, Esin Akbay Çetin, Mehmet Ali Onur, Selis Önel, Ali Tuncel

**Affiliations:** † Bioengineering Division, Hacettepe University, Ankara 06800, Turkey; ‡ Graduate School of Science & Engineering, Hacettepe University, Ankara 06800, Turkey; § Department of Biology, Hacettepe University, Ankara 06800, Turkey; ∥ Chemical Engineering Department, Hacettepe University, Ankara 06800, Turkey

**Keywords:** nanozyme, peroxidase-like activity, Fenton-like reaction, glutathione depletion, chemodynamic therapy

## Abstract

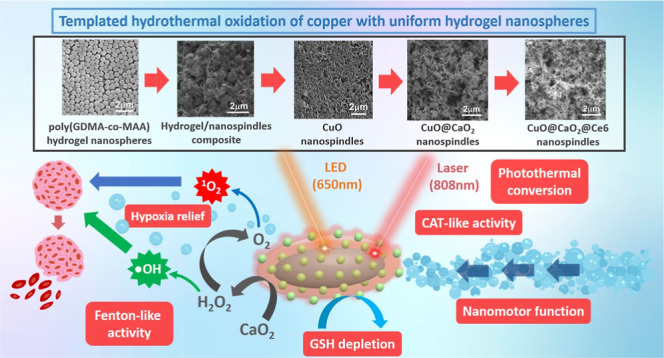

Uniform, mesoporous copper(II) oxide nanospindles (CuO NSs) were synthesized via a method based on templated hydrothermal oxidation of copper in the presence of monodisperse poly(glycerol dimethacrylate-*co*-methacrylic acid) nanoparticles (poly(GDMA-*co*-MAA) NPs). Subsequent decoration of CuO NSs with a CaO_2_ nanoshell (CuO@CaO_2_ NSs) yielded a nanozyme capable of Cu(I)/Cu(II) redox cycling. Activation of the Cu(I)/Cu(II) cycle by exogenously generated H_2_O_2_ from the CaO_2_ nanoshell significantly enhanced glutathione (GSH) depletion. CuO@CaO_2_ NSs exhibited a 2-fold higher GSH depletion rate compared to pristine CuO NSs. The generation of oxygen due to the catalase (CAT)-like decomposition of H_2_O_2_ by CuO@CaO_2_ NSs resulted in a self-propelled diffusion behavior, characteristic of a H_2_O_2_ fueled nanomotor. These nanostructures exhibited both peroxidase (POD)-like and CAT-like activities and were capable of self-production of H_2_O_2_ in aqueous media via a chemical reaction between the CaO_2_ nanoshell and water. Usage of the self-supplied H_2_O_2_ by the POD-like activity of CuO@CaO_2_ NSs amplified the generation of toxic hydroxyl (^•^OH) radicals, enhancing the chemodynamic effect within the tumor microenvironment (TME). The CAT-like activity provided a source of self-supplied O_2_ via decomposition of H_2_O_2_ to alleviate hypoxic conditions in the TME. Under near-infrared laser irradiation, CuO@CaO_2_ NSs exhibited photothermal conversion properties, with a temperature elevation of 25 °C. The combined GSH depletion and H_2_O_2_ generation led to a more effective production of ^•^OH radicals in the cell culture medium. The chemodynamic function was further enhanced by an elevated temperature. To assess the therapeutic potential, CuO@CaO_2_ NSs loaded with the photosensitizer, chlorine e6 (Ce6), were evaluated against T98G glioblastoma cells. The synergistic combination of photodynamic, photohermal, and chemodynamic modalities using CuO@CaO_2_@Ce6 NSs resulted in cell death higher than 90% under in vitro conditions.

## 1 Introduction

Nanomaterials with enzyme-like properties, the so-called nanozymes, have been used in diverse applications such as biomedical diagnosis and treatment, sensing of chemicals/biochemicals, and chemical and photochemical catalysis.^1^ The oxidoreductase-like, hydrolase-like, and isomerase-like activities have been obtained using nanozymes mostly in the form of inorganic nanomaterials.^1^ A more stable chemical structure and function under various conditions can be achieved using nanozymes instead of natural enzymes. These nanozymes have shown great potential in cancer therapy, offering a synergistic approach by combining multiple therapeutic modalities, such as chemotherapy, immunotherapy, radiotherapy, chemodynamic therapy, photodynamic therapy, and photothermal therapy.^2,3^

CuO-based composite nanoplatforms with different chemical structures and morphologies have been evaluated as nanozymes for sensing of various biochemicals such as ascorbic acid, alkaline phosphatase, 3,4-dihydroxyphenylalanine enantiomers, epinephrine, glucose, and dopamine.^4−10^ CuO-based nanozymes were used as an electrochemical cytosensor for sensitive detection of circulating tumor cells and various chemicals such as hydroquinone and hexavalent chromium.^11−13^ CuO-based nanozymes also exhibited antibacterial activity and evaluated for bacterial killing and wound disinfection.^14−16^

Copper-based nanozymes have multiple enzyme mimetic behaviors such as peroxidase (POD)-like, catalase (CAT)-like, and superoxide dismutase-like activities. The facile synthesis, facile elimination in the biological media, low cytotoxicity, and the production ability of toxic hydroxyl (^•^OH) radicals are the noticeable superiorities making copper-based nanozymes suitable nanomaterials in different biological applications. Due to all of these properties, copper-based nanozymes have attracted considerable attention as synergistic therapy agents.

The combinatorial effects of copper(II) oxide nanoparticles (CuO NPs) on MCF-7 cells were examined under hyperthermia and irradiation.^17^ A separate group of therapy applications were performed by loading CuO-based nanoplatforms with different antineoplastic agents. A folic acid-attached alginate polydopamine-modified Zn–CuO nanocomposite loaded with paclitaxel, disulfiram/mitoxantrone-loaded mesoporous CuO NPs, and PHBV@polydopamine coated CuO NPs loaded with 5-fluorouracil and paclitaxel were evaluated for synergistic therapy of different tumors.^18−20^ The nanocomposites based on the combination of CuO NPs with different metal oxides such as ZnO, CeO_2_, and NiO were also loaded with different antitumoral agents and successfully used for various combinatorial therapy applications.^21−24^

Cu(I) ions released from a copper oxide-based nanoplatform react with already found or self-supplied H_2_O_2_ in TME to produce highly toxic ^•^OH radicals according to the Fenton-like mechanism.^25−27^ Glutathione (GSH) depletion ability is the other important superiority of CuO-based nanoplatforms used in synergistic therapy applications.^28−31^ The formation of Cu(I) ions by the reduction of released Cu(II) cations with GSH already found in the tumor microenvironment (TME) triggers the generation of ^•^OH radicals via a Fenton-like mechanism. Owing to these properties, copper(II) oxide-based nanoplatforms have recently gained considerable attention particularly in chemodynamic therapy (CDT).^28−31^

The combination of CaO_2_ nanoparticles (CaO_2_ NPs) with different nanoplatforms allows for the synthesis of synergistic therapy agents, which are capable of ROS amplification and hypoxia relief by the Fenton-like and CAT-like activities of selected nanoplatforms, respectively.^32−37^ Various copper-based nanostructures such as a copper-containing hybrid organosilica framework, CuS NPs, and Cu-5,10,15,20-tetrakis(4-carboxyphenyl)porphyrin complex were combined with CaO_2_ NPs for obtaining therapy agents suitable for CDT via Fenton-like activity and by amplifying ROS in TME.^38−41^

In this study, a new method was developed for the synthesis of a new nanomaterial: mesoporous copper(II) oxide nanospindles (CuO NSs). The nanoplatform exhibited a photothermal conversion ability under near-infrared (NIR) laser irradiation. CuO NSs were coated with a thin CaO_2_ nanoshell. The resulting CaO_2_ nanoshell-attached CuO nanospindles [CuO@CaO_2_ NSs] exhibited POD-like and CAT-like activities and were able to generate H_2_O_2_ in aqueous medium. The usage of self-supplied H_2_O_2_ via POD-like activity of CuO@CaO_2_ NSs allowed for the production of ^•^OH radicals, which in turn activates chemodynamic action in TME. CuO@CaO_2_ NSs were self-propelled nanomotors capable of converting self-supplied H_2_O_2_ into a mechanical movement via O_2_ generation by their CAT-like activity. The usability of the synthesized nanozyme as a potential therapy agent with photothermal, photodynamic, and chemodynamic functions was exemplified by the interaction of CuO@CaO_2_ NSs loaded with a clinically approved photosensitizer, chlorine e6 (Ce6), using T98G glioblastoma cells under in vitro conditions.

## 2 Experimental Section

### 2.1 Materials

All chemicals used for preparation of poly(glycerol dimethacrylate-*co*-methacrylic acid) nanoparticles, (poly(GDMA-*co*-MAA) NPs) and CuO NSs were supplied from Sigma-Aldrich, USA. Chlorin e6 (Ce6) was obtained from Medkoo, USA. Copper(II) chloride, calcium chloride (CaCl_2_), tris(hydroxymethyl)aminomethane, acetic acid, sodium acetate, hydrochloric acid (HCl, 37.5% w/w), 3,3′,5,5′-tetramethylbenzidine (TMB), *o*-phenylenediamine (OPDA), hydrogen peroxide (50% w/w), 1,3-diphenylisobenzofuran (DPBF), titanium(IV) chloride (TiCl_4_), ethanol, and acetonitrile (ACN) were purchased from Sigma-Aldrich. Dulbecco’s modified Eagle’s medium (DMEM)/Ham’s F12 and fetal bovine serum (FBS) were supplied from Biochrom AG, Berlin, Germany. T98G human glioblastoma cells and L929 subcutaneous connective tissue cells were obtained from American Type Culture Collection (ATCC, VA, USA). Dimethyl sulfoxide (DMSO), phosphate buffered saline (PBS), 0.25% trypsin–EDTA, acridine orange (AO), propidium iodide (PI), 2,7-dichlorofluorescein diacetate (DCFDA), 3-(4,5-dimethylthiazol-2-yl)-2,5-diphenyl tetrazolium bromide (MTT), and penicillin/streptomycin (P/S) were supplied from Sigma-Aldrich. All synthesis runs and cell culturing experiments were conducted using deionized (DI) water with a resistivity of 18 MΩ cm (Direct-Q3, Millipore, U.S.A).

### 2.2 Characterization of Spindle-like Nanoparticles

The porous properties of CuO NSs and CuO@CaO_2_ NSs were determined by the nitrogen physisorption method using the Brunauer–Emmett–Teller model in the surface area and pore size analyzer (Quantachrome, Nova 2200E, UK). The size distribution, the mean particle size, and the surface morphology of CuO NSs and CuO@CaO_2_@Ce6 NSs were analyzed by scanning electron microscopy (SEM, Tescan, Czech Republic). The morphology and shell structure of CuO@CaO_2_ NSs were analyzed by transmission electron microscopy (TEM, Technai, FEI, Thermo Fisher Scientific, Oregon, U.S.A.). The crystalline structure was evaluated by X-ray diffraction (XRD) spectrophotometer (Rigaku Ultima-IV, Japan). The surface chemistry of spindle like-nanoparticles was investigated by X-ray photoelectron spectroscopy (XPS, K-Alpha XPS system, Thermo Fischer Scientific, USA). Cu and Ca contents of CuO@CaO_2_ NSs were analyzed by optically coupled plasma–optical emission spectrophotometry (ICP–OES, 720 ES, Varian/Agillent, U.S.A.).

### 2.3 Synthesis of CuO NSs

CuO NSs were synthesized using a shape-templated hydrothermal protocol for the first time. For this purpose, uniform cross-linked-poly(glycerol dimethacrylate-*co*-methacrylic acid) nanoparticles [poly(GDMA-*co*-MAA) NPs] were used as the template. Poly(GDMA-*co*-MAA) NPs were prepared by precipitation polymerization.^42^ Typically, glycerol dimethacrylate (GDMA) (1.5 mL) was copolymerized with methacrylic acid (MAA) (0.65 mL) using AIBN (0.16 g) as the initiator in a continuous medium containing toluene (42.3 mL) and ACN (22.7 mL) at 70 °C for 24 h in a Pyrex-sealed glass reactor.^42^

For the synthesis of CuO NSs, CuCl_2_·2H_2_O (200 mg) was dissolved in DI water (40 mL) and poly(GDMA-*co*-MAA) NPs (100 mg) were added to the solution. The dispersion was stirred at 300 rpm at room temperature. After 2 h, hexamethylenetetramine (HMTA, 400 mg in 4.0 mL DI water) was injected and the oxidation reaction was conducted at 75 °C for 6 h under magnetic stirring at 300 rpm. CuO/polymethacrylate composite nanoparticles were collected by centrifugation and washed with water, using a centrifugation/decantation protocol. After drying of composite nanoparticles at 70 °C for overnight, CuO NSs were obtained by removing the polymethacrylate template from the composite nanoparticles by calcination at 550 °C for 2 h with a ramp of 2 °C/min.^43^

### 2.4 Preparation of CuO@CaO_2_ NSs

CuO (50 mg) NSs were well dispersed in methanol by ultrasonication. CaCl_2_ solution (2 M, 0.5 mL) was introduced to the CuO/methanol mixture, and the resulting medium was vigorously stirred at room temperature for 2 h. NH_4_OH solution (2 M, 1 mL) was added to the dispersion. Then, H_2_O_2_ solution (0.5 mL, 50% w/w) was fed using a syringe pump in 10 min, and the medium was stirred for 10 min at room temperature. The final product, CuO@CaO_2_ NSs, was washed with anhydrous methanol by using a centrifugation/decantation protocol and redispersed in anhydrous methanol by ultrasonication for 2 min^44^

### 2.5 Preparation of Ce6-Loaded CuO@CaO_2_ NSs

For the adsorption of Ce6 onto CuO@CaO_2_ NSs, CuO@CaO_2_ NSs (5.0 mg) were added into Ce6-methanol solution (1.0 mg/mL, 1 mL) and the obtained dispersion was stirred for 30 min at room temperature. The obtained CuO@CaO_2_@Ce6 NSs were washed with methanol by centrifugation/decantation. To determine Ce6 adsorption onto CuO@CaO_2_ NSs, CuO@CaO_2_@Ce6 NSs were separated by centrifugation at 5000 rpm for 10 min, and the absorbance of the supernatant originated from the unbound Ce6 in the solution was measured at 400 nm in a UV–vis spectrophotometer (Thermo Scientific, Genesys 150, USA). The equilibrium Ce6 binding onto CuO@CaO_2_ NSs (*Q*_Ce6_) is calculated according to eq 1, where *A*_o_ and *A*_f_ are the absorbances of the initial Ce6-methanol solution and the supernatant, respectively. *C*_Ce6_ (mg/mL) is the initial concentration of Ce6 in methanol, *V* (mL) is the volume of adsorption medium, and *M*_NS_(g) is the mass of CuO@CaO_2_ NSs.^45^
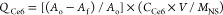
1

### 2.6 GSH Depletion by CuO and CuO@CaO_2_ NSs

The GSH depletion was determined using 5,5′-dithiobis(2-nitrobenzoic acid) (DTNB). GSH (1 mM) was added to the aqueous dispersion of CuO or CuO@CaO_2_ NSs (2.0 mg/mL, 10 mL), and the medium was shaken at 37 °C at a rate of 100 cpm in the dark. The sample taken from the dispersion (1.0 mL) was centrifuged, and the supernatant (500 μL) was collected. DTNB solution (50 μL, 10 mM) and PBS buffer (2.5 mL) were added to the supernatant. The absorbance of resulting solution was measured at 412 nm in a UV–vis spectrophotometer (Thermo Scientific, USA).^46,47^ GSH depletion runs with CuO NSs and CuO@CaO_2_ NSs also were also performed with lower concentrations of both NSs (i.e., 0.1 and 0.2 mg/mL) to explain the mechanism of GSH depletion.

Intracellular GSH depletion was also determined by using DTNB as the probe. T98G cells were seeded in 6-well plates at a density of 4 × 10^4^ cells/well. The cells were incubated with CuO NSs or CuO@CaO_2_@Ce6 NSs for 60 min by changing the NS concentration in the range of 0.05–2.0 mg/mL. Then, trypsin was added to detach the cells from the surface of the well. The cells were centrifuged at 800 rpm for 5 min and washed with PBS three times. The cells were lysed using Triton-X-100 buffer. The medium was centrifuged at 10,000 rpm for 20 min, and the supernatant was collected. A certain volume of supernatant (50 μL) was mixed with DTNB solution (50 μL, 10 mM). The absorbance of resulting solution was measured by a microplate reader (μQuantTM, BiotekW Instruments Inc., USA).^48^

### 2.7 Hydrogen Peroxide Generation by CuO@CaO_2_ NSs

TiCl_4_ was used as the probe for the determination of H_2_O_2_ generated by CuO@CaO_2_ NSs in media at pH 5 and 7. For this purpose, CuO@CaO_2_ NSs (2 mg/mL) were dispersed in an acetate buffer (pH 5.0, 50 mM) or a tris buffer (pH 7.0, 50 mM). The reaction for H_2_O_2_ evolution was carried out at room temperature in the dark with a rotating speed of 100 rpm. The samples were collected at designated times and centrifuged for the separation of the CuO@CaO_2_ NSs. After the supernatant was collected, a TiCl_4_ solution (1 mL, 84 mM) was added. The concentration of H_2_O_2_ generated in the solution by CuO@CaO_2_ NSs was determined by absorbance measurement at 414 nm in a UV–vis a spectrophotometer (Thermoscientific, Genesys 150, USA).^45^

### 2.8 Monitoring of Hydroxyl Radical Generation

The production of ^•^OH radicals was detected by using fluorescence spectroscopy using 2,5-dihydroxyterephthalic acid (DHTPA) as the fluorescent probe. Briefly, CuO or CuO@CaO_2_ NSs (12.0 mg) were dispersed in an acetate buffer (pH 5.0, 50 mM, 12 mL) containing terephthalic acid (TPA, 5 mM) and H_2_O_2_ (5 mM). The medium was stirred for 2 h at 350 rpm and 37 °C in the dark for the formation of DHTPA by the reaction between TPA and ^•^OH radicals generated in the acidic medium. After 2 h, the NSs were collected from the aqueous phase by centrifugation at 5000 rpm. The fluorescence spectrum of the supernatant was recorded with an excitation wavelength of 315 nm in a fluorescence spectrophotometer (Varian, Cary Eclipse, USA).^45^ The same run was also performed at a higher temperature (i.e., 45 °C) using CuO@CaO_2_ NSs under the same conditions to see the effect of temperature on the production rate of ^•^OH radicals.^45^

### 2.9 POD-like and Oxidase (OD)-like Activities of CuO@CaO_2_ NSs

For POD-like activity, CuO NSs or CuO@CaO_2_ NSs (2.0 mg/mL) were dispersed in tris buffer (50 mM, pH 7.0). OPDA solution (150–3000 μM, 6 mL) and H_2_O_2_ solution (2 μL, 50% w/w) were added to the obtained dispersion, and the medium was shaken at room temperature. The samples were withdrawn from the reaction medium at prescribed times and centrifuged for the separation of NSs from the liquid part. The absorbance of the supernatant was recorded at 416 nm in a UV–vis spectrophotometer (Thermo Scientific, Genesys 150, USA). Initial consumption rates with different OPDA concentrations were calculated using the expression given in an earlier work.^45,49^

On the other hand, by considering the self-supplied H_2_O_2_ generated by the reaction between CaO_2_ nanoshell on CuO NSs and water, the self-POD-like activity of CuO@CaO_2_ NSs was also determined. The protocol for the determination of the self-POD-like activity of CuO@CaO_2_ NSs was identical with the one followed for the POD-like activity of CuO@CaO_2_ NSs except no H_2_O_2_ addition was made in this assay. The self-POD-like activity of CuO@CaO_2_ NSs was investigated using OPDA at pH 7.0 and TMB at pH 5.0.^45,49^

The OD-like activity was not studied for CuO@CaO_2_ NSs due to the self-supplied H_2_O_2_ generated by the CaO_2_ nanoshell on the CuO NSs. OD-like activity of CuO NSs was determined according to the following protocol. Typically, CuO NSs (2.0 mg/mL) were dispersed in an acetate buffer (50 mM, pH 5.0). The dispersion was shaken at a rate of 100 cpm in the dark at 37 °C. TMB stock solution (50–500 μM, 6 mL) was then added to the dispersion. The samples were withdrawn from the reaction medium and centrifuged at 5000 rpm for 5 min. After the separation of NSs, the absorbance of the supernatant was measured at 650 nm. Initial consumption rates with different TMB concentrations were calculated using the expression given in an earlier work.^45,49^

### 2.10 CAT-like Activity of CuO NSs

CAT-like activity of CuO NSs was determined by monitoring the decomposition of H_2_O_2_ using a colorimetric protocol.^50^ Typically, CuO NSs (2.0 mg/mL) were dispersed in an aqueous H_2_O_2_ solution (0.1–1 mM) and the medium was magnetically stirred at room temperature. The samples withdrawn from the reaction medium at prescribed times were centrifuged at 5000 rpm for 5 min. 1 mL of TiCl_4_ solution was then added to 1 mL of the supernatant. The absorbance of the TiCl_4_/H_2_O_2_ complex was determined at 414 nm in a UV–vis spectrophotometer. The initial H_2_O_2_ consumption rate was calculated based on the variation of complex concentration with the time for the initial reaction period as described elsewhere.^50^

### 2.11 Photodynamic Properties of CuO@CaO_2_@Ce6 NSs

The photodynamic response of CuO@CaO_2_@Ce6 NSs was evaluated by using diphenylbenzofuran (DPBF) as a singlet oxygen probe.^45^ Typically, CuO@CaO_2_@Ce6 NSs (5.0 mg/mL) were dispersed in DPBF solution (30 μg/mL in acetonitrile), and the resulting dispersion was irradiated with a LED at 650 nm wavelength (0.8 W). The samples were taken from the dispersion at various times. The absorption spectra of supernatants obtained by centrifugation of the samples were recorded in a UV–vis spectrophotometer at room temperature. Only the DPBF solution, and the aqueous dispersions containing only CuO and only CuO@CaO_2_ NSs were used as controls.^45^

### 2.12 Photothermal Properties of CuO@CaO_2_@Ce6 NSs

The aqueous dispersions were prepared at different CuO@CaO_2_@Ce6 NS concentrations ranging between 0.05 and 2.0 mg/mL. The aqueous dispersion of CuO@CaO_2_@Ce6 NSs (200 μL) was irradiated using an NIR laser at 808 nm for 5 min with a power density of 1.3 W/cm^2^. Temperature elevation was recorded by using a thermocouple. For the observation of thermal stability, the aqueous dispersion of CuO@CaO_2_@Ce6 NSs was tested under five cycles with 808 nm laser irradiation.^45,50^

### 2.13 Interaction of CuO@CaO_2_@Ce6 NSs with T98G Cells via Photothermal, Photodynamic, and Chemodynamic Effects

Human glioblastoma cells (T98G) were used as the model tumor cell and cultured in a DMEM medium containing 10% (w/w) FBS and 1% (w/w) P/S at 37 °C in a 5% (v/v) CO_2_ atmosphere. The cells were seeded in 96-well plates at a density of 2 × 10^4^ cells/well as in the previous study.^45^ The cells were treated with CuO and CuO@CaO_2_@Ce6 NSs with different concentrations alone to see the cytotoxic effect of NSs in the absence of light irradiation. Then, CuO@CaO_2_@Ce6 NSs were incubated at different concentrations (0.05–2.0 mg/mL) with T98G cells. The cells were irradiated with red LED at 650 nm for 7 min and followed by 808 nm NIR laser for 5 min, and then the cells were incubated at 37 °C for 24 h. The cell viability was determined using the MTT assay. The culture medium was replaced with the fresh medium containing MTT (200 μL, 10% w/w MTT), and the cells were incubated for 4 h at 37 °C. Isopropyl alcohol was replaced to dissolve the generated formazan crystals. The optical density was measured at 570 nm by a microplate reader (μQuantTM, BiotekW Instruments Inc., USA).^45^

Using the AO/PI dual cell staining method, the cells with a density of 2 × 10^4^ cells/well were cultivated in 96-well plates to track the cell death caused by the synergistic approach. LED and NIR applications were performed according to the above procedure. After LED and NIR applications, the cells were incubated 24 h at 37 °C. Before imaging, the cell medium was replaced with the dye solution (100 μL, 1:1, v/v) and incubated for 2 min. The cells were washed with PBS twice and examined using an inverted fluorescence microscope (Olympus IX70, Japan). A control run with CuO NSs was conducted under the same conditions, but without the use of an LED or an NIR laser, to observe any potential cytotoxic effect.

In vitro cytotoxicity of CuO NSs was also investigated using the L929 mouse fibroblast cell line. L929 cells were cultured in DMEM-F12 medium containing 10% w/w FBS and 1% w/w P/S at 37 °C under a 5% (v/v) CO_2_ atmosphere. L929 cells were seeded into 96-well culture plates with a cell density of 2 × 10^4^ cells/well. The cells were incubated with CuO NSs at different concentrations (0.05–2.0 mg/mL). The viability of L929 cells was determined using MTT assay, and live/dead cell images were obtained by dual cell staining with the AO/PI system as also described above for T98G cells.^45^

### 2.14 Detection of Intracellular ROS with CuO@CaO_2_@Ce6 NSs

For the monitoring of intracellular ROS generation by CuO@CaO_2_@Ce6 NSs, DCFDA was used as the ROS probe. Typically, T98G cells were seeded with a cell density of 2 × 10^4^ cells/well and CuO@CaO_2_@Ce6 NSs were introduced into the cell suspension at a concentration of 0.25 mg/mL. Then DCFDA (10 mM) was solubilized in the resulting dispersion. The cells were irradiated using an NIR laser at 808 nm and a red LED at 650 nm for 5 and 7 min, respectively. As a control run, the aqueous dispersion only containing T98G cells and CuO@CaO_2_@Ce6 NSs was also followed without applying NIR laser/LED irradiation. The fluorescent emission originated from intracellular ROS generation was observed under fluorescence microscope (Olympus, IX70, Japan).^45^

### 2.15 TUNEL Assay

The cells were seeded in 96-well plates at a density of 2 × 10^4^ cells/well, as described above. Apoptotic cells were identified using the terminal deoxynucleotidyl Transferase-mediated dUTP Nick End Labeling (TUNEL) assay. The cell death was assessed using the DNA fragments formed in the late stages of apoptosis. Cells were treated with CuO@CaO_2_@Ce6 NSs (0.1 and 1.0 mg/mL) and irradiated with a red LED for 7 min and a NIR laser for 5 min. After a 24 h incubation at 37 °C, cells were fixed with 1% w/w paraformaldehyde solution. Subsequently, the ApopTag Peroxidase In Situ Apoptosis Detection Kit (Chemicon, Millipore) was used to stain apoptotic cells, following the manufacturer’s protocol. Cell images were captured using an optical microscope.^51^

### 2.16 Scratch Assay for Cell Proliferation and Migration

T98G cells were seeded in a 6-well plate at a density of 2 × 10^5^ cells/well and cultured for 24 h. Once 70–80% confluency was reached, a 200 μL sterile pipet tip was used to create scratch wounds on the cell monolayer. Cells were washed twice with PBS and treated with CuO@CaO_2_@Ce6 NSs at concentrations of 0.1, 0.5, and 1.0 mg/mL. Subsequently, cells were irradiated with a 650 nm LED for 7 min and an 808 nm NIR laser for 5 min. Cells were then incubated at 37 °C in DMEM supplemented with 0.5 μg/mL allantoin and 10% (w/w) FBS that served as the positive control, while serum-free medium was used as the negative control. Cell proliferation and migration were monitored at 6, 24, and 36 h using an inverted microscope. At the 36 h time point, crystal violet staining was performed to assess wound closure and cell migration potential. The culture medium was removed, and the cells were fixed with 3.7% w/w paraformaldehyde solution, permeabilized with methanol for 20 min, and stained with 1% w/w crystal violet solution. After double-washing with PBS, the stained cells were examined using an inverted microscope.^52^

## 3 Results and Discussion

### 3.1 Morphological and Chemical Characterization of NSs

**Figure 1:**
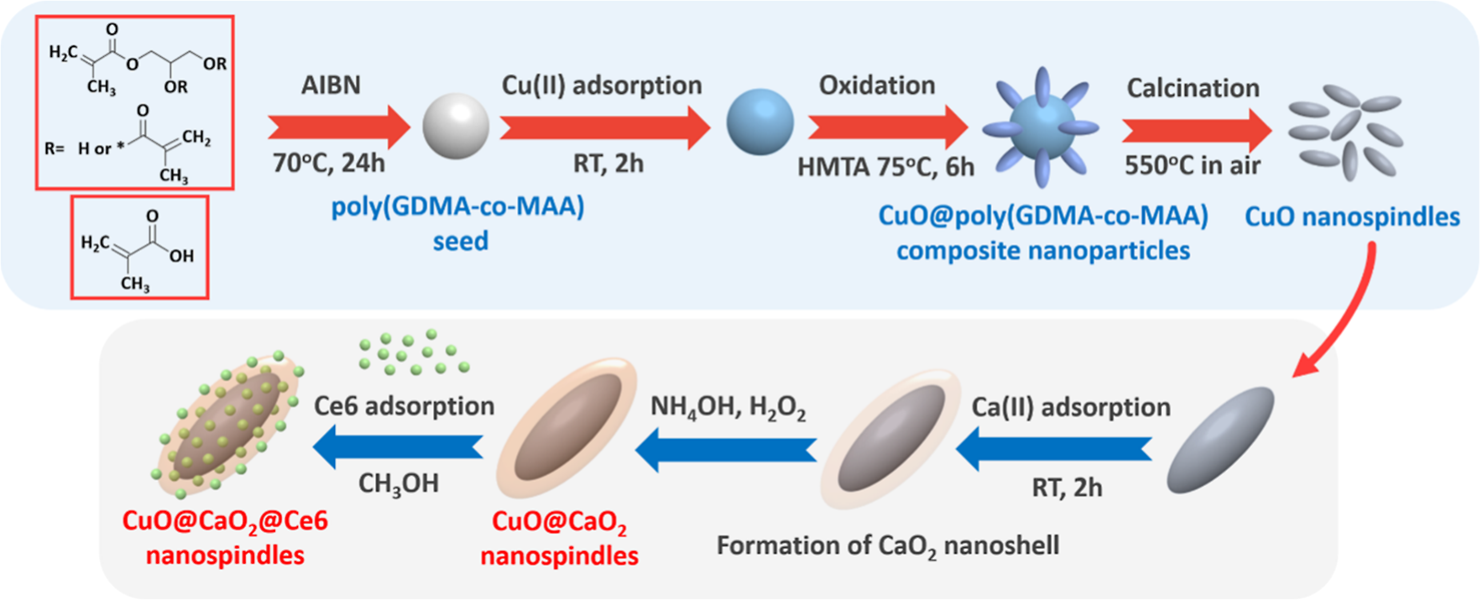
Schematic representation of the chemical route used for the synthesis of CuO@CaO_2_@Ce6 NSs.

A new hydrothermal route was proposed for the templated hydrothermal synthesis of CuO NSs. The schematic representation of the preparation protocol of CuO@CaO_2_ NSs is given in Figure 1.

**Figure 2:**
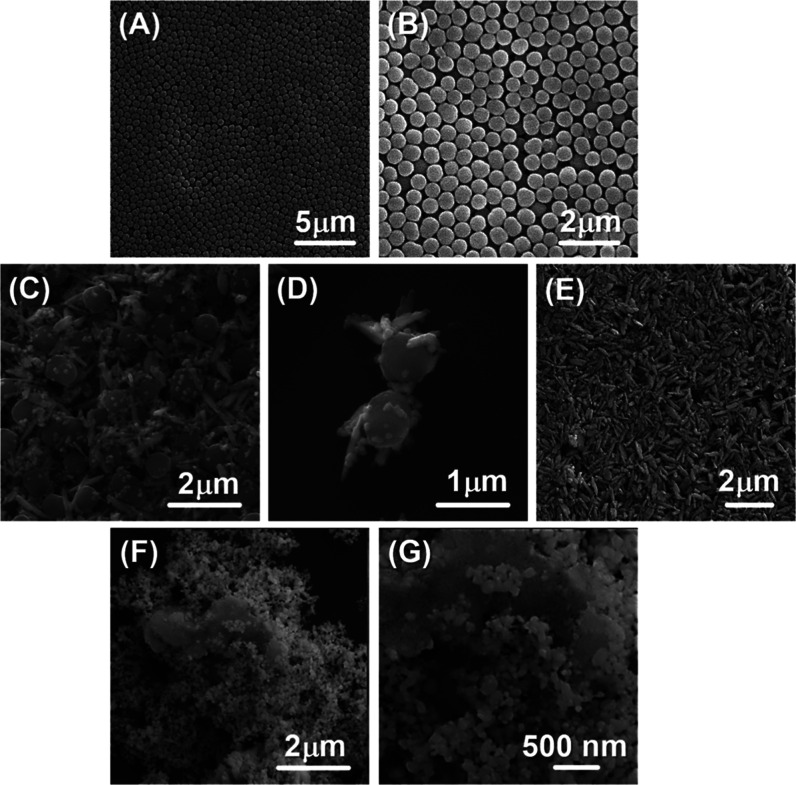
SEM photographs of poly(GDMA-*co*-MAA) NPs obtained by precipitation polymerization used as a template in the synthesis of CuO NSs. Magnification: (A) 10.0 and (B) 25.0 KX. (C,D) SEM photographs of Cu(OH)_2_/polymethacrylate composite nanoparticles. Magnification: 30.0 and 60.0 KX for (C,D), respectively. (E) SEM photograph of CuO NSs synthesized in the presence of poly(GDMA-*co*-MAA) NPs. Magnification: 20.1 KX. (F,G) SEM photographs of CuO NSs synthesized in the absence of poly(GDMA-*co*-MAA) NPs. Magnification: 30.0 and 70.0 KX for (F,G), respectively.

Here, uniform-cross-linked poly(GDMA-*co*-MAA) NPs obtained by precipitation polymerization was used as the template to obtain pristine CuO NSs with a narrow size distribution.^42^ A typical SEM photograph of poly(GDMA-*co*-MAA) NPs is included in Figure 2A,B. In the first stage, Cu(II) cations were adsorbed onto poly(GDMA-*co*-MAA) NPs via the complexation with carboxyl groups. The formation of Cu(OH)_2_/polymethacrylate composite nanoparticles was achieved by the chemical reaction taking place on poly(GDMA-*co*-MAA) NPs, conducted using HMTA as the oxidation agent. The SEM photographs of Cu(OH)_2_/polymethacrylate composite nanoparticles are given in Figure 2C,D. As seen here, the Cu(OH)_2_ phase was grown on uniform poly(GDMA-*co*-MAA) NPs mostly in the form of nanorods. Pristine CuO NSs were obtained by the calcination of Cu(OH)_2_/polymethacrylate composite nanoparticles in an air atmosphere at 550 °C for the removal of the polymeric phase. The SEM photograph of CuO NSs synthesized by using poly(GDMA-*co*-MAA) NPs as the template is given in Figure 2E. A SEM photograph with higher magnification is also included in Figure S1A of the Supporting Information. The synthesis performed by using poly(GDMA-*co*-MAA) NPs as nucleation sites provided regular CuO NSs with a narrow size distribution (Figure 2E). In the shape-templated process, the initiation of the spindle-like nanoparticle formation process via physically adsorbed Cu(II) cations onto poly(GDMA-*co*-MAA) NPs should be the factor providing the formation of regular CuO NSs. The SEM photographs of pristine CuO NSs synthesized in the absence of poly(GDMA-*co*-MAA) NPs are also included in Figure 2F,G.

Here, CuO NSs were obtained with a broad size distribution, containing large, irregular microparticles in the absence of poly(GDMA-*co*-MAA) NPs. The presence of large irregular microparticles should be due to the initiation of a smaller number of nuclei in the absence of heterogeneous nucleation sites and enhanced growth during the formation of the Cu(OH)_2_ phase within the continuous medium.^53,54^ Poly(GDMA-*co*-MAA) NPs exhibited a high equilibrium Cu(II) adsorption [i.e., 408.0 mg Cu(II)/g poly(GDMA-*co*-MAA)] in an adsorption period of 2 h, due to the complex formation with the carboxyl groups of NPs (Figure S2 of the Supporting Information). In other words, 55.1% of Cu(II) initially loaded into the synthesis medium of CuO NSs was adsorbed onto poly(GDMA-*co*-MAA) NPs before the addition of oxidation agent (i.e., HMTA). The preferential localization of Cu(II) ions on the surface of poly(GDMA-*co*-MAA) NPs supports the formation of nucleation predominantly on the surface of the NPs, leading to the generation of well-defined CuO NSs.

Following the synthesis of regular pristine CuO NSs with narrow size distribution using poly(GDMA-*co*-MAA) NPs as the template, a CaO_2_ nanoshell was generated on CuO NSs via the chemical reaction between CaCl_2_ and H_2_O_2_ in the aqueous dispersion containing CuO NSs (Figure 1). Then the photosensitizer Ce6 was loaded onto CuO@CaO_2_ NSs via physical adsorption in methanolic solution.

**Figure 3:**
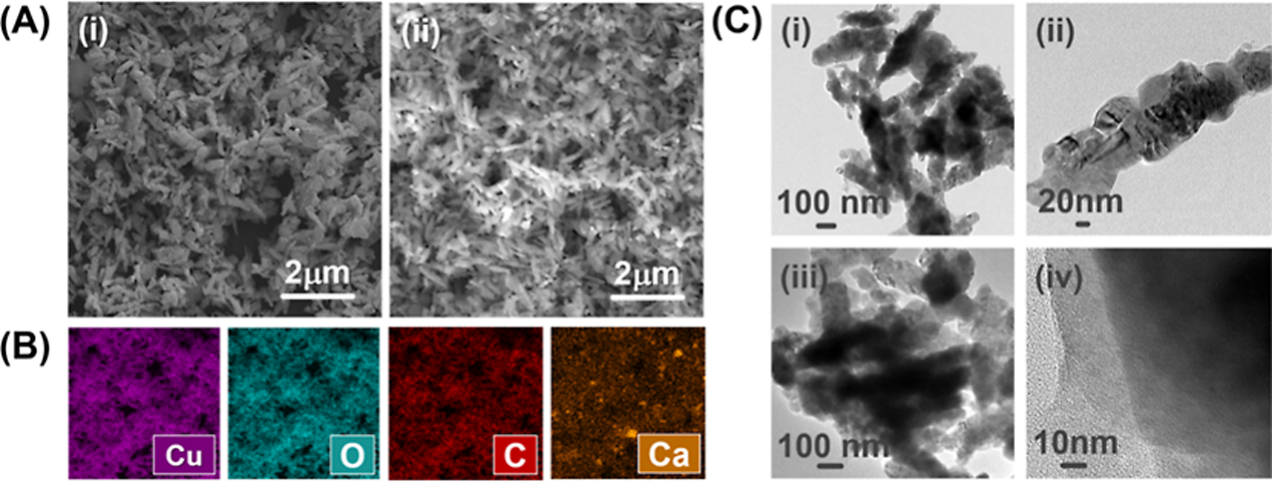
(A) SEM photographs of CuO@CaO_2_ and CuO@CaO_2_@Ce6 NSs: NS type and magnification: (i) CuO@CaO_2_, 25.0 KX, (ii) CuO@CaO_2_@Ce6, 25.1 KX, (B) EDX spectroscopy images of CuO@CaO_2_ NSs. (C) TEM photographs of CuO and CuO@CaO_2_ NSs. NS type: (i,ii) CuO, (iii) CuO@CaO_2_, and (iv) CaO_2_ nanoshell on CuO NSs. The scale bars are given on the photographs.

The SEM photographs of CuO@CaO_2_ and CuO@CaO_2_@Ce6 NSs are also presented in Figure 3A(i,ii), respectively. No significant change occurred in the dimensions and shape of CuO NSs by the formation of the CaO_2_ nanoshell (Figure 3A). The attachment of Ce6 to CuO@CaO_2_ NSs also caused no significant change in the morphological properties of CuO@CaO_2_ NSs (Figure 3A). The SEM photographs of CuO@CaO_2_ and CuO@CaO_2_@Ce6 NSs with higher magnifications are also given in Figure S1B,C of the Supporting Information, respectively.

The size characteristics of CuO and CuO@CaO_2_ NSs are given in Table 1. As seen here, the mean length values of CuO and CuO@CaO_2_ NSs were determined as 499 and 539 nm, according to the SEM photographs given in Figures 2E and 3A(i), respectively. The approximate mean diameters of CuO and CuO@CaO_2_ NSs were calculated as 135 and 141 nm, respectively, based on the same photographs. Hence, the approximate aspect (i.e., length/diameter) ratios of CuO and CuO@CaO_2_ NSs were found to be 3.70 and 3.82, respectively.

**Table 1:** Size and Porous Properties of CuO and CuO@CaO_2_@Ce6 NSs^a^

sample	*L*_avg_ (nm)	*D*_avg_ (nm)	average aspect ratio (*L*_avg_/*D*_avg_)	SSA (m^2^/g)	pore volume (cm^3^/g)	mean pore diameter (nm)
CuO	499	135	3.70	18.20	0.046	25.9
CuO@CaO_2_@Ce6	539	141	3.82	33.9	0.056	26.0

a *L*_avg_: average length, *D*_avg_: average diameter, SSA: specific surface area.

Energy-dispersive X-ray spectroscopy (EDX) images of CuO@CaO_2_ NSs and CuO NSs are given in Figures 3B and S3, respectively. Cu, O, Ca, and C atoms were observed on both CuO and CuO@CaO_2_ NSs. The presence of C in CuO NSs should likely originate from the calcined polymethacrylate template. The TEM photographs of CuO and CuO@CaO_2_ NSs are shown in Figure 3C. Pristine CuO NSs had an irregular, rugged surface morphology [Figure 3C(i,ii)]. No significant change occurred in the shape of CuO NSs by the formation of a CaO_2_ nanoshell around them (Figure 3C(iii)). The thickness of the CaO_2_ nanoshell on CuO NSs was roughly 10 nm, as shown in Figure 3C(iv).

**Figure 4:**
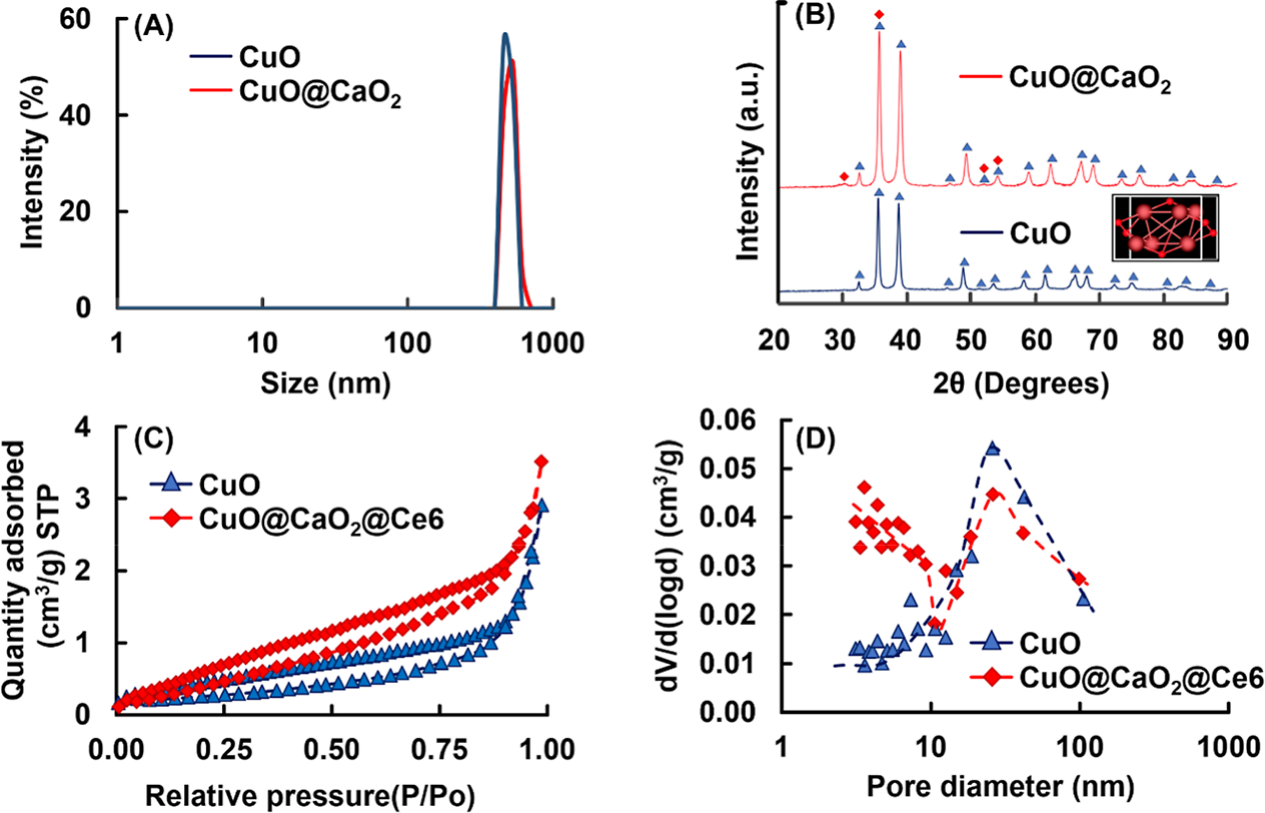
(A) Hydrodynamic size distributions, (B) XRD patterns, (C) nitrogen adsorption–desorption isotherms, and (D) pore size distribution curves of CuO and CuO@CaO_2_ NSs.

The hydrodynamic size distributions of CuO and CuO@CaO_2_ NSs determined by dynamic light scattering in DI water are given in Figure 4A. The median hydrodynamic size values of CuO and CuO@CaO_2_ NSs were determined as 458.7 and 531.2 nm, respectively. The higher mean hydrodynamic size of CuO@CaO_2_ NSs is explained by the formation of a CaO_2_ nanoshell on CuO NSs. XRD patterns of CuO and CuO@CaO_2_ NSs are given in Figure 4B. The XRD patterns of both NSs exhibited a crystalline structure, represented by sharp, clear peaks. The XRD pattern of pristine CuO NSs suited to the monoclinic crystal structure of tenorite. The peaks at 2θ angles of 32.49, 35.51, 38.67, 46.24, 48.72,51.38, 53.44, 56.73, 58.13, 61.54, 66.28, 68.10, 72.42, 74.95, 80.14, 82.75, 86.49, and 89.79° were assigned to (1 1 0), (0 0 2), (1 1 1), (1 1–2), (2 0–2), (1 1 2), (0 2 0), (0 2 1), (2 0 2), (1 1–3), (3 1–1), (1 1 3), (3 1 1), (2 2–2), (1 1–4), (2 2 2), (2 2–3), and (1 3–1) planes of the monoclinic crystal structure, respectively (ICDD 04-008-8209).^55,56^

The calculated structural parameters for the lattice constants (*a*, *b*, *c*), the unit cell volume (*V*), the average crystallite size (*D*_savg_), and *d*-spacing values of CuO NSs are given in Table 2.

**Table 2:** Structural Parameters of CuO NSs

	lattice parameters				*V* (Å^3^) (*a*, *b*, *c*, sin β)	*D*_sAvg_ (nm)	*d*-spacing (Å)
NSs	*a* (Å)	*b* (Å)	*c* (Å)	β			
CuO	4.70	3.43	5.14	99.57	81.74	18.31	1.61

For CuO@CaO_2_ NSs, sharp peaks belonging to the tenorite phase were also observed. The peaks specific to CaO_2_ phase were also detected at 2θ values of 30.2, 35.5, and 51.5, 53.5° (ICDD 03-0865).^44,57,58^ The Ca/Cu atomic ratio in the bulk structure of CuO@CaO_2_ NSs was determined as 0.172 by ICP-OES spectroscopy.

The nitrogen adsorption isotherms and the pore size distribution curves of CuO and CuO@CaO_2_ NSs are given in Figure 4C,D, respectively. Type (IV) adsorption isotherms were obtained with both NSs, indicating their mesoporous structures containing the pores in the range 3–100 nm. An appreciable fraction of low-sized pores at ca. 3 nm was observed by the attachment of the CaO_2_ nanoshell onto CuO NSs. The porous properties of CuO and CuO@CaO_2_ NSs are presented in Table 1. Based on the pore-size distributions given in Figure 4D, the specific surface areas of CuO and CuO@CaO_2_ NSs were determined as 18.2 and 33.9 m^2^/g, respectively (Table 1). The increase in the surface area should be explained by the formation of a CaO_2_ nanoshell as an additional porous compartment on the CuO NSs.

Survey XPS spectra of pristine CuO and CuO@CaO_2_@Ce6 NSs are given in Figure S4 of the Supporting Information. The surface atomic compositions calculated from Survey XPS spectra are given in Table S1. In the survey XPS of CuO NSs, Cu 2p, O 1s, and C 1s peaks were obtained at 934.1, 530.1, and 285.1 eV, respectively (Figure S3). In the survey XPS spectrum of CuO@CaO_2_@Ce6 NSs, Cu 2p, O 1s, and C 1s peaks were observed at the binding energies of 934.1, 532.1, and 285.1 eV, respectively (Figure S3). Additionally, Ca 2p and N 1s peaks were obtained at binding energies of 348.1 and 398.1 eV due to the CaO_2_ nanoshell and Ce6 immobilized on CuO@CaO_2_ NSs, respectively. A C 1s peak with a higher intensity with respect to CuO NSs was also obtained for CuO@CaO_2_@Ce6 NSs likely due to the immobilization of Ce6 on CuO@CaO_2_ NSs.

**Figure 5:**
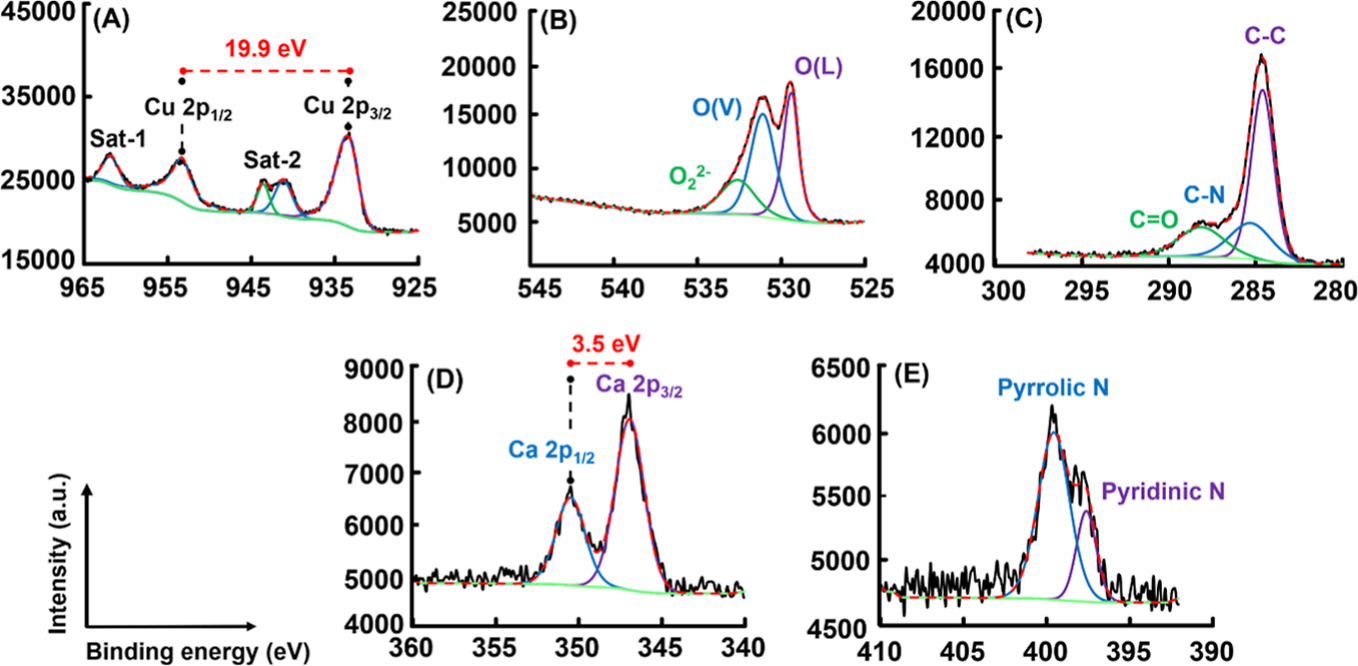
XPS of CuO@CaO_2_@Ce6 NSs. Core level spectra for (A) Cu 2p scan, (B) O 1s scan, (C) C 1s scan, (D) Ca 2p scan, and (E) N 1s scan.

The core level spectra for Cu 2p scan for CuO@CaO_2_@Ce6 NSs are given in Figure 5A. The core level spectra for Cu 2p, O 1s, and C 1s scan of CuO NSs are presented in Figure S5 of the Supporting Information. Cu 2p spectra of both NSs were deconvoluted into four peaks, which included two types of spin–orbit lines called satellite peaks (Sat-1 and Sat-2) at the binding energies of 961.9 and 943.3–940.9 eV. The peaks confirming the presence of the Cu(II) valence state in the CuO phase of CuO@CaO_2_@Ce6 NSs were observed at the binding energies of 933.6 and 953.3 eV belonging to Cu 2p_3/2_ and Cu 2p_1/2_ levels, respectively (Figure 5A). The same peaks belonging to the Cu(II) valence state on CuO NSs were also obtained with small shifts lower than 0.5 eV at the corresponding binding energy values (Figure S5A). The binding energy difference of ca. 19.7 eV between these peaks confirmed the presence of CuO phase in both NSs (Figures 5A and S5A).^43,55,59,60^

In the core level spectra for O 1s scan of CuO@CaO_2_@Ce6 NSs and pristine CuO NSs, three main peaks were detected (Figures 5B and S5B of the Supporting Information). The peak located at ca. 529.4 eV in O 1s spectra of CuO@CaO_2_@Ce6 NSs was assigned to lattice oxygen [O(L)] (Figure 5B).^61,62^ In the same spectra, the peak at 531.1 eV was assigned to oxygen vacancy.^61,62^ The peaks belonging to O(L) and O(V) of the peaks of the CuO NSs appeared at binding energy values slightly shifted with respect to those obtained for the peaks of the CuO@CaO_2_@Ce6 NSs (Figure S5B). The peak positioned at 532.6 eV in the O 1s spectra of CuO@CaO_2_@Ce6 NSs was due to the O_2_^2–^ valence state coming from CaO_2_ on CuO@CaO_2_@Ce6 NSs (Figure 5B).^63,64^ In the O 1s spectra of pristine CuO NSs, the deconvoluted peak at 533.2 eV should be due to the adsorbed oxygen (Figure S5B of the Supporting Information).^65^

The core level spectra for C 1s scan with CuO@CaO_2_@Ce6 NSs is given in Figure 5C. The deconvoluted peaks ca. at 284.6 and 285.3 eV in the C 1s spectra of CuO@CaO_2_@Ce6 NSs are associated with the C–C and C–N bonds, respectively (Figure 5C).^66^ In the C 1s spectra of CuO NSs, the deconvoluted peaks at 284.7 and 286.2 eV were assigned to C–C and C–O bonds, respectively (Figure S5C).^67^

The core level spectra for Ca 2p scan for CuO@CaO_2_@Ce6 NSs are given in Figure 5D. The presence of a Ca 2p peak is an indicator for the formation CaO_2_ nanoshell on the outer surface of CuO NSs. The binding energies of Ca 2p_3/2_ and Ca 2p_1/2_ peaks were obtained at 346.9 and 350.4 eV, respectively. The peak-to-peak separation between Ca 2p_3/2_ and Ca 2p_1/2_ levels was 3.5 eV, indicating that Ca(II) was in the oxidized state in the corresponding phase.^57,59^ The peaks obtained at 397.6 and 399.6 eV in the core level spectra for N 1s scan of CuO@CaO_2_@Ce6 NSs were assigned to pyridinic nitrogen and pyrrolic nitrogen, respectively, originated from Ce6 immobilized on CuO@CaO_2_ NSs (Figure 5E).^68,69^

### 3.2 Nanomotor Function of CuO@CaO_2_ NSs

**Figure 6:**
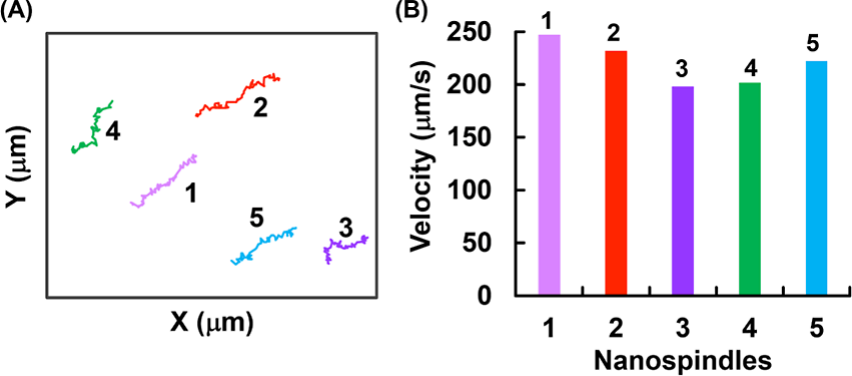
(A) Trajectory of the motion originated from the nanomotor function of CuO@CaO_2_ NSs on XY plane for different NS clusters. (B) Velocity of different NSs.

The movement of CuO@CaO_2_ spindle-like nanomotors in the aqueous medium containing H_2_O_2_ was observed under an inverted optical microscope (Olympus, IX73, Tokyo, Japan). The H_2_O_2_ fueled-convective motion of CuO@CaO_2_ spindle-like nanomotors were exemplied by Movies S1, S2 and S3 of the Supporting Information. Two different aqueous dispersions containing CuO@CaO_2_ spindle-like nanomotors at low (1.0 mg/mL) and high (15.0 mg/mL) concentrations were used. In the aqueous dispersion containing CuO@CaO_2_ NSs at low concentration, the movement of individual spindle-like nanoparticles was observed with the small oxygen bubbles generated and then bursted around them (Movie S1 of the Supporting Information). The generation of O_2_ bubbles by the decomposition of H_2_O_2_ (i.e., marked by red circles using CapCut software on Movie S1) via the CAT-like activity is the driving force for the convective motion (i.e., self-propelled diffusion) of CuO@CaO_2_ spindle-like nanomotors (Movie S1). In this run, additional H_2_O_2_ was used with an initial concentration of about 100 mM to follow the bubble formation clearly. The trajectories of small NS clusters were demonstrated by a blue tracking line on Movie S2, using Fiji/ImageJ software. These trajectories confirmed that the motion of CuO@CaO_2_ NSs was different from the conventional Brownian motion. The trajectory of the motion originated from the nanomotor function of CuO@CaO_2_ NSs on the XY plane is shown for different NS clusters in Figure 6A. The velocities of different NS clusters followed on Movie S2 are shown in Figure 6B. The high velocities ranging between 200 and 250 μm/s originated from the self-propelled diffusion of CuO@CaO_2_ NSs were calculated for six different nanospindle clusters, as shown in Movie S2.

Note that following the motion of individual NSs with the mean length/mean diameter ratio of 539 nm/141 nm is difficult under an optical microscope. For this reason, the oxygen evolution was clearly observed in the form of large bubbles together with the evident motion of microaggregates in the concentrated dispersion of CuO@CaO_2_ NSs (15.0 mg/mL) (Movie S3 of the Supporting Information). The convective motion of CuO@CaO_2_ clusters containing a large number of NSs was observed in the form of gray clouds moving away from the microaggregates (i.e., marked by red circles using CapCut software on Movie S3). Here, a concentrated dispersion of CuO@CaO_2_ NSs containing H_2_O_2_ with a high concentration (i.e., 1.0 M) was used to observe the generation of O_2_ bubbles and also the convective motion of CuO@CaO_2_ clusters (i.e., microaggregates) under an optical microscope. The self-propelled diffusion of CuO@CaO_2_ clusters was a convective motion created by the generation and the expansion of O_2_ bubbles by the CAT-like activity of CuO@CaO_2_ NSs. The momentum produced by the expansion of an O_2_ bubble is transferred to CuO@CaO_2_ clusters placed on the bubble surface. Then, the self-propelled diffusion of CuO@CaO_2_ clusters started to move away from the surface of growing O_2_ bubbles (Movie S3).^70,71^

The comparison of Movies S1 and S3 clearly demonstrated that the number and growth rate of the O_2_ bubbles increased with the increasing concentration of CuO@CaO_2_ NSs and increasing concentration of H_2_O_2_. The nanomotor function can be evaluated as a factor positively contributed to the enhancement of interaction between CuO@CaO_2_ NSs and tumor cells in TME. In order to test this hypothesis, CuO NSs and CuO@CaO_2_ NSs (0.5 mg/mL) were interacted with nonadherent T98G cell suspension without or with 1 mM H_2_O_2_ for 10 min. In each case, the interaction of both NSs with the cells was examined under an inverted microscope with ×400 magnification in phase contrast mode. The effect of the nanomotor function on the interaction of CuO and CuO@CaO_2_ NSs with T98G cells is shown in Figure S6 of the Supporting Information. In principle, the nanomotor function of both NSs should be enhanced by adding H_2_O_2_ into the cell culture medium, due to the generation of O_2_ bubbles via their CAT-like activity. The image of T98G cells in the control medium containing no NS and no H_2_O_2_ is given in Figure S6A. The number of CuO or CuO@CaO_2_ NSs interacted with the cells were relatively lower in the absence of H_2_O_2_ (Figure S6B,C). As seen in Figure S6D,E, a higher number of CuO and CuO@CaO_2_ NSs were attached onto the cells in the presence of 1 mM H_2_O_2_. Particularly, the number of NSs that interacted with T98G cells was considerably higher with respect to all other cases in the medium containing both CuO@CaO_2_ NSs and H_2_O_2_. The hypothesis on the enhancement of the interaction between NSs and T98G cells by their nanomotor function was supported by the micrographs given in Figure S6.^72,73^

### 3.3 GSH Depletion by CuO and CuO@CaO_2_ NSs

The sequential reaction scheme that is expected to take place in the presence of CuO@CaO_2_ NSs is given by eqs 2–5. The generation of H_2_O_2_ by CaO_2_ nanoshell by the reaction given by eq 2 allows for the reproduction of Cu^2+^ ions via the reaction in eq 4 and then additional GSH depletion according to eq 3.^38,74−76^ GSH acting an antioxidant to capture ROS in TME is overexpressed in tumor cells.^77^ For this reason, the depletion of GSH allows for the amplification of ROS in TME. Cu(II) ions released from CuO@CaO_2_ NSs are reacted with GSH to produce Cu(I) ions and glutathione disulfide (GSSG) [eq 3].^74,76^ The Fenton-like reaction can efficiently take place in TME at pH between 6.5 and 6.9 when it is catalyzed by Cu(I) ions and also is much faster than that by Fe(II), which provides faster ROS generation [eq 4].^78^ In other words, toxic ^•^OH radicals are efficiently produced in a weakly acidic or neutral medium by the Fenton-like reaction activated by Cu(I) ions.^31,38,74^ Therefore, the coexistence of GSH and H_2_O_2_, as an “AND” logic gate should activate CuO@CaO_2_@Ce6 NSs to perform the copper-assisted chemodynamic therapy on T98G cells.^76^ Ultimately, GSH depletion and generation of toxic ^•^OH radicals result in DNA damage, inactivation of proteins, peroxidation of lipids, and ultimately cell apoptosis.^76^ On the other hand, some of the generated H_2_O_2_ are decomposed for oxygen production via CAT-like activity of CuO@CaO_2_@Ce6 NSs for enhancing the effect of PDT applied using ^1^O_2_ radicals generated from Ce6 immobilized on CuO@CaO_2_ NSs [eq 5].
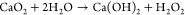
2
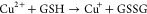
3
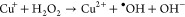
4


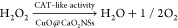
5

**Figure 7:**
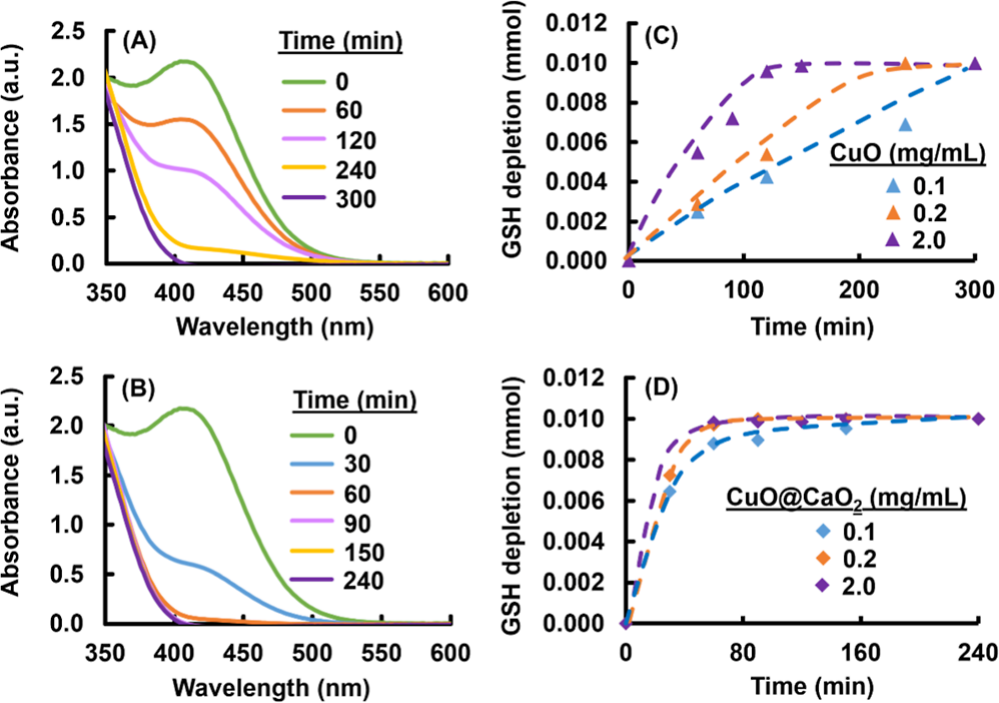
Sample UV–vis spectra recorded for GSH depletion with (A) CuO NSs and (B) CuO@CaO_2_ NSs using DTNB as the reactive probe. Concentration of CuO and CuO@CaO_2_ NSs: 0.2 mg/mL. The variation of GSH depleted with the time by (C) CuO NSs and (D) CuO@CaO_2_ NSs at different concentrations. GSH initial concentration: 1 mM, reaction volume: 10 mL, temperature: 37 °C.

Representative UV–vis spectra obtained at different times for the GSH depletion media including CuO and CuO@CaO_2_ with a constant NS concentration of 0.2 mg/mL are given in Figure 7A,B, respectively. On the other hand, GSH depletion was investigated by changing the NS concentration between 0.1 and 2.0 mg/mL. This concentration range was also employed in in vitro experiments with T98G cells to evaluate the PDT, PTT, and CDT potentials of CuO@CaO_2_@Ce6 NSs. The UV–vis spectra obtained with the NS concentrations of 0.1 and 2.0 mg/mL at different times are also included for both CuO and CuO@CaO_2_ NSs in Figure S7 of the Supporting Information. The GSH depletion profiles obtained with CuO and CaO_2_ NSs at different NS concentrations are shown in Figure 7C,D, respectively.

The main findings obtained in the GSH depletion runs are listed below:

I For a certain concentration of NSs, GSH depletion is about two times faster with CuO@CaO_2_ NSs with respect to that obtained with pristine CuO NSs (Figure 7C,D).
II Complete depletion of GSH (1 mM) was achieved with either pristine CuO or CuO@CaO_2_ NSs at all concentrations of these nanomaterials (Figure 7C,D). The complete GSH depletion with all concentrations of CuO@CaO_2_ NSs demonstrated that Cu(II) cations released from both types of NSs participated into the GSH reduction reaction given by eq 3 and the Cu(I) cations formed in this reaction were then converted to Cu(II) by the reaction given in eq 4. The combination of the behavior by the generation of self-supplied H_2_O_2_ via CaO_2_ nanoshell described in Cu(I) formed a Cu(I)/Cu(II) regeneration cycle in the presence of CuO@CaO_2_ NSs.
III As seen in Figure 7C,D, the GSH depletion rate is a function of NS concentration for both pristine CuO and CuO@CaO_2_ NSs. The GSH depletion rate increased with the increasing NS concentration for either pristine CuO and CuO@CaO_2_ NSs. The increase occurred in the GSH depletion rate with the NS concentration was more appreciable with CuO NSs (Figure 7C). The GSH depletion rates obtained with all concentrations of CuO@CaO_2_ NSs were already high, and a gradual increase which was similar to CuO NSs was not observed for CuO@CaO_2_ NSs (Figure 7D). This finding was an indicator of the superior GSH depletion ability of CuO@CaO_2_ NSs with respect to pristine CuO NSs. The regeneration of Cu(II)/Cu(I) cycle shown by eqs 2 and 3 by means of H_2_O_2_ produced by CaO_2_ nanoshell immobilized on CuO NSs should be positively contributed to the superior GSH depletion ability of CuO@CaO_2_ NSs.

The impact of NS concentration on intracellular GSH levels for CuO and CuO@CaO_2_ NSs is illustrated in Figure S8 of the Supporting Information. Intracellular GSH was effectively depleted by both CuO and CuO@CaO_2_ NSs and the depletion increased with the increasing concentration of both NSs. CuO@CaO_2_ NSs provided higher GSH depletion with respect to that observed with CuO NSs due to the generation of additional H_2_O_2_ by the CaO_2_ nanoshell.^79,80^ More effective depletion of intracellular GSH by NSs makes weaker the defense mechanism of tumor cells by increasing the ROS concentration and the oxidative damage.^81^

### 3.4 ROS Generation with CuO@CaO_2_ and CuO@CaO_2_@Ce6 NSs

**Figure 8:**
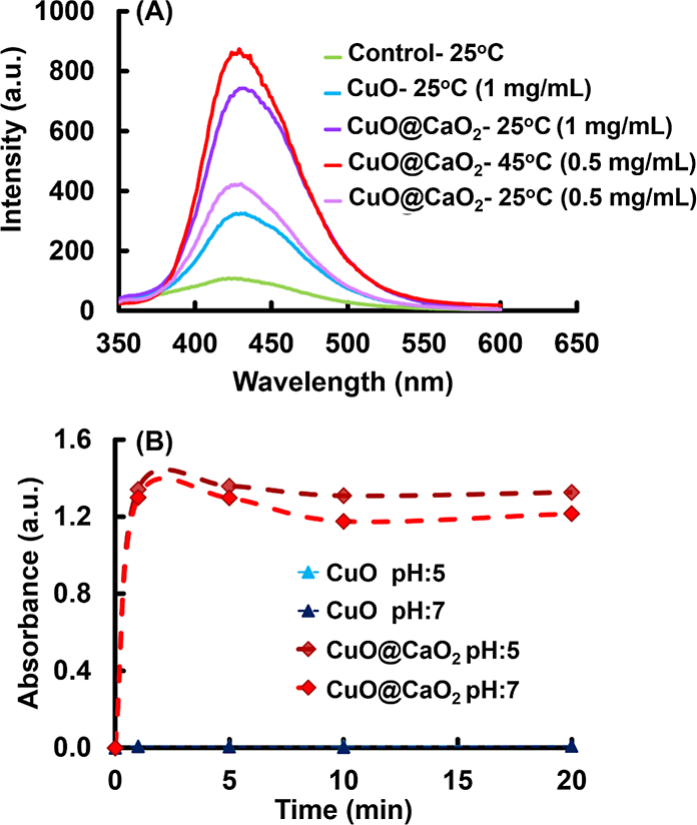
(A) Fluorescence spectra showing the generation of ^•^OH radicals by CuO and CuO@CaO_2_ NSs, excitation: 315 nm, emission: 430 nm, fluorescent probe: 2,5-DHTPA. The concentrations of CuO and CuO@CaO_2_ NSs: 0.5 and 1.0 mg/mL. Temperature: 25 or 45 °C. (B) Generation of H_2_O_2_ by CuO@CaO_2_ NSs. CuO@CaO_2_ concentration: 2.0 mg/mL.

The production of ^•^OH radicals was monitored by fluorescence spectroscopy using DHTPA as a fluorescent probe. The fluorescence spectra recorded with CuO and CuO@CaO_2_ NSs are given in Figure 8A. When the ^•^OH radical generation runs performed at room temperature, the maximum emission at 445 nm proportional to the concentration of 2,5-DHTPA was higher for CuO@CaO_2_ NSs with respect to that obtained with CuO NSs. The formation of additional H_2_O_2_ due to the chemical reaction between the CaO_2_ nanoshell on CuO NSs should result in the generation of a higher number of ^•^OH radicals via the Fenton-like activity reaction, which in turn results in the formation of –DHTPA with a higher concentration. Note that the fluorescence intensity at 445 nm markedly increased when the concentration of CuO@CaO_2_ NSs was elevated from 0.5 to 1.0 mg/mL at room temperature due to the production of a higher number of ^•^OH radicals. On the other hand, when the fluorescence spectra obtained with CuO@CaO_2_ NSs at room temperature and 45 °C with the NS concentration of 0.5 mg/mL were compared, a serious increase in the fluorescence intensity was observed with the increasing temperature. This comparison confirmed that the number of ^•^OH radicals was increased by elevating the temperature (Figure 8A).

The production of H_2_O_2_ by the reaction between CaO_2_ nanoshell on CuO NSs and H_2_O (i.e., eq 2) was demonstrated by the determination of H_2_O_2_ concentration in the aqueous medium via complex formation with TiCl_4_. As seen in the plot showing the variation of H_2_O_2_ concentration with time, the generation of H_2_O_2_ by CuO@CaO_2_ NSs in the aqueous medium was very fast and completed almost in 1 min (Figure 8B).

**Figure 9:**
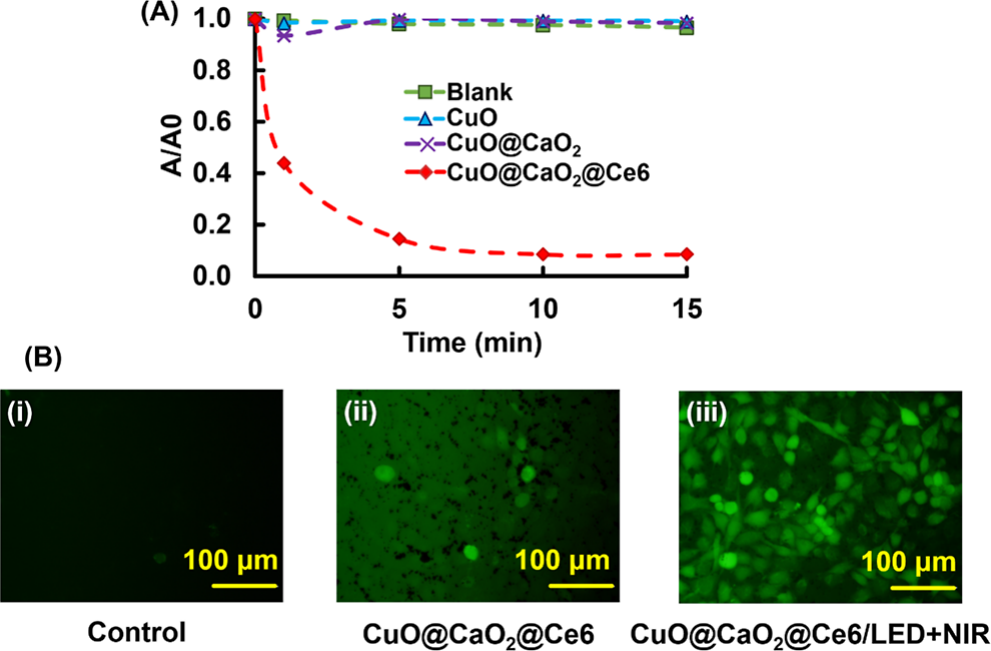
ROS generation behaviors of CuO@CaO_2_ and CuO@CaO_2_@Ce6 NSs. (A) ^1^O_2_ radical generation by CuO@CaO_2_ and CuO@CaO_2_@Ce6 NSs. Probe: DPBF, concentration of CuO@CaO_2_ and CuO@CaO_2_@Ce6 NSs: 5.0 mg/mL. (B) Intracellular ROS formation with CuO@CaO_2_ and CuO@CaO_2_@Ce6 NSs with T98G cells. (i) Control, (ii) CuO@CaO_2_@Ce6 NSs, and (iii) CuO@CaO_2_@Ce6 with NIR laser@808 nm for 5 min + LED@650 nm irradiation for 7 min. T98G cell concentration: 2 × 10^4^ cells/well, concentration of CuO@CaO_2_ and CuO@CaO_2_@Ce6 NSs: 0.25 mg/mL.

The generation of ^1^O_2_ radicals by CuO@CaO_2_ NSs was followed by the decrease in the absorbance of DPBF at 417 nm due to the consumption of DPBF by ^1^O_2_ radicals generated in the aqueous dispersions containing CuO, CuO@CaO_2_ and CuO@CaO_2_@Ce6 NSs irradiated by visible light at 650 nm (Figure 9A). As seen here, no significant change occurred in the absorbance of the aqueous media containing CuO or CuO@CaO_2_ NSs since no significant ^1^O_2_ radical production took place in the absence of visible light. However, the absorbance rapidly decreased in 5 min due to the consumption of DPBF by ^1^O_2_ radicals generated by Ce6 immobilized on CuO@CaO_2_ NSs under visible light irradiation.

Ce6 was loaded onto CuO@CaO_2_ NSs by physical adsorption in methanol. The variation of equilibrium Ce6 adsorption onto CuO@CaO_2_ NSs with the initial Ce6 concentration is given in Figure S9A of the Supporting Information. Based on these results, the Ce6 initial concentration of 1 mg/mL was selected as the appropriate value for loading Ce6 onto CuO@CaO_2_ NSs. This resulted in a Ce6 content of 104.7 mg of Ce6/g of CuO@CaO_2_ NSs in the final composite material. The cumulative release of Ce6 from CuO@CaO_2_@Ce6 NSs in phosphate buffer media at pH 5.5 and 7.0 is given in Figure S9B of the Supporting Information. The release of Ce6 is faster at pH 5.5 with respect to that observed at neutral pH. Faster dissolution of CaO_2_ nanoshell at acidic pH is likely the reason for the observed behavior.^79^ However, the behavior in Figure S9B demonstrates that >90% of initially loaded Ce6 is found on CuO@CaO_2_@Ce6 NSs during the time periods used for in vitro synergistic interactions (i.e., red LED irradiation for 7 min in photodynamic effect and NIR laser irradiation for 5 min in the phothermal effect).

The fluorescence microscope photographs taken with the cell culture media containing T98G cells and different concentrations of CuO@CaO_2_@Ce6 NSs are presented in Figure 9B. No significant fluorescence was observed with the control medium containing only T98G cells (Figure 9B(i)). A weak green fluorescence emission was observed in the cell culture medium containing CuO@CaO_2_@Ce6 NSs upon excitation at 485 nm. This fluorescence is likely due to the generation of ^•^OH radicals via a Fenton-like reaction triggered by the production of H_2_O_2_ from the CaO_2_ nanoshell on CuO@CaO_2_@Ce6 NSs in an aqueous solution at 37 °C [Figure 9B(ii)]. The strongest fluorescence intensity was obtained when the cell culturing medium containing CuO@CaO_2_@Ce6 NSs was irradiated with both NIR laser@808 nm and LED@650 nm (Figure 9B(iii)). The generation of ^1^O_2_ radicals by Ce6 adsorbed onto CuO@CaO_2_ NSs was triggered by visible light irradiation. On the other hand, the chemical reactions for generation of H_2_O_2_ by CaO_2_ nanoshell and the generation of ^•^OH radicals via Fenton-like activity of CuO NSs were accelerated by the temperature elevation induced by the photothermal conversion ability of CuO NSs under NIR laser irradiation. Hence, an appreciable elevation occurred in the formation of intracellular ROS when T98G cell culturing medium containing CuO@CaO_2_@Ce6 NSs was subjected to irradiation by an LED and NIR laser. Figure 9B(iii) demonstrates that CuO@CaO_2_@Ce6 NSs is a potential therapeutic agent capable of producing ^•^OH and ^1^O_2_ radicals in the T98G cell culturing medium via CDT and PDT modalities, respectively.

### 3.5 POD-like, OD-like, and CAT-like Activities of NSs

**Figure 10:**
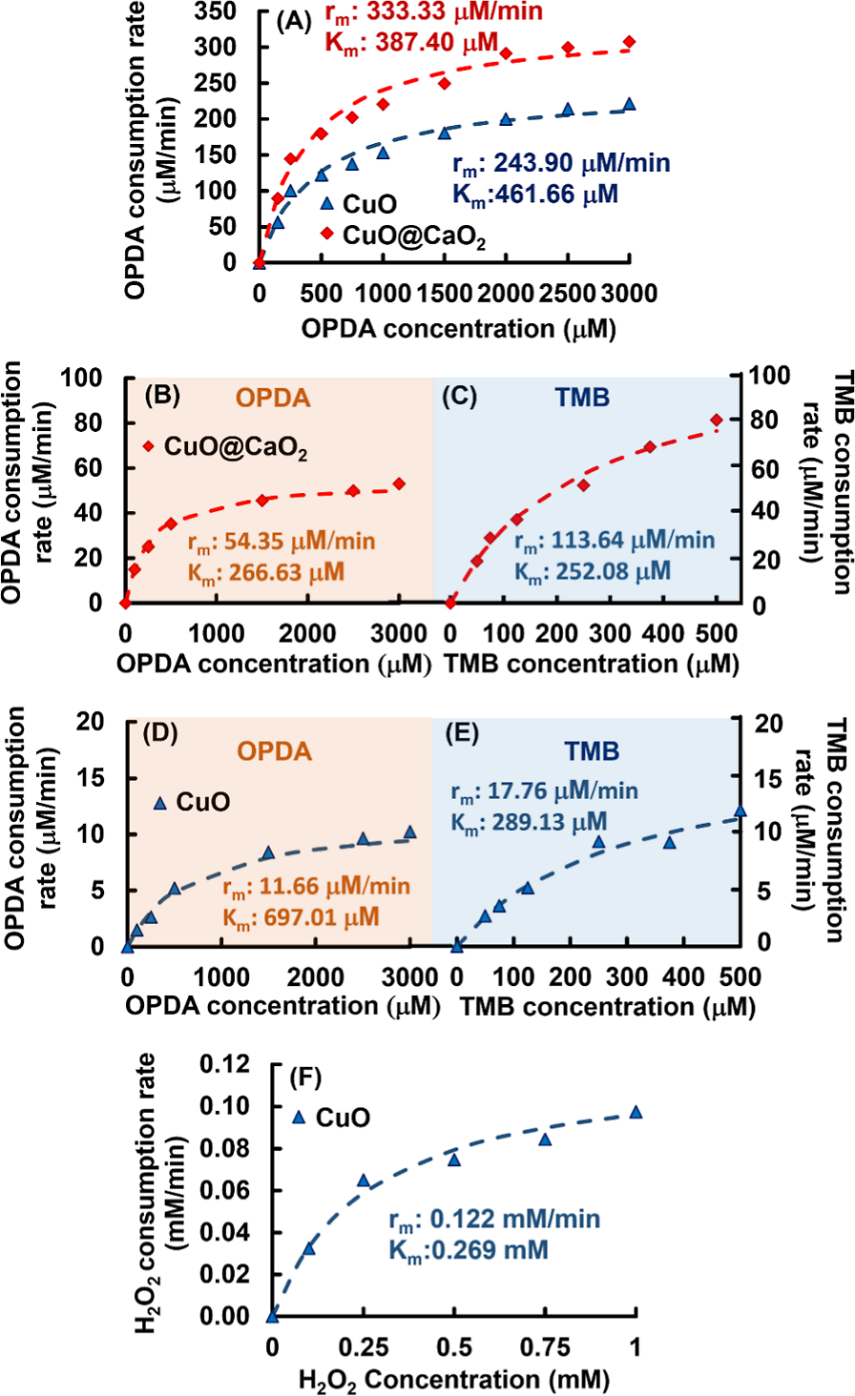
Michaelis–Menten plots for (A) POD-like activities of CuO and CuO@CaO_2_ NSs, (B) self-POD-like activity of CuO@CaO_2_ NSs without adding H_2_O_2_ using OPDA as the substrate at pH 7.0, (C) self-POD-like activity of CuO@CaO_2_ NSs without adding H_2_O_2_ using TMB as the substrate at pH 5.0, (D) OD-like activity of CuO NSs using OPDA as the substrate at pH 7.0, (E) OD-like activity of CuO NSs using TMB as the substrate at pH 5.0, and (F) CAT-like activity of CuO NSs. Nanozyme concentration: 2.0 mg/mL. Temperature: 25 °C.

The Michaelis–Menten plot for the POD-like activity of CuO and CuO@CaO_2_ NSs using OPDA as the substrate in the colorimetric reaction taking place at pH 7.0 is given in Figure 10A. High maximum substrate consumption rates obtained with CuO and CuO@CaO_2_ NSs indicated that both nanostructures exhibited strong POD-like activity. The substrate consumption rates with CuO@CaO_2_ NSs were higher with respect to those found with CuO NSs when OPDA was used as the substrate at pH 7.0. In the POD-like activity assay performed with CuO@CaO_2_ NSs under these conditions, the final concentration of H_2_O_2_ in the colorimetric reaction medium was higher with respect to that used with CuO NSs since a certain extent of H_2_O_2_ was already generated via the CaO_2_ nanoshell on CuO NSs. Hence, the colorimetric conversion of OPDA at pH 7.0, using CuO@CaO_2_ NSs as the nanozyme was conducted with higher H_2_O_2_ concentration which in turn is higher POD-like activity with CuO@CaO_2_ NSs (Figure 10A). The self-POD-like activity of CuO@CaO_2_ NSs was also determined without adding H_2_O_2_ in the colorimetric conversion of OPDA at pH 7.0 (Figure 10B). In this run, only the H_2_O_2_ generated by the CaO_2_ nanoshell was used in the colorimetric reaction. As expected, a lower maximum substrate consumption rate (i.e., 54.35 μM/min) was obtained with respect to that found by using externally added H_2_O_2_ into the reaction medium for intrinsic POD-like activity of CuO@CaO_2_ NSs at pH 7.0, using OPDA as the substrate (i.e., 333.3 μM/min) (Figure 10A,B). The previous research revealed that the H_2_O_2_ formation rate in the reaction between CaO_2_ and water was higher in the acidic pH with respect to the neutral medium.^79,82^ By considering this finding, another substrate (i.e., TMB) whose optimum pH in the colorimetric conversion via POD-like activity was different from that of OPDA was selected. Hence, the self-POD-like activity of CuO@CaO_2_ NSs was investigated using TMB as the substrate at pH 5.0 (Figure 10C). Higher TMB conversion rates with different TMB initial concentrations, approximately 2-fold higher maximum TMB conversion rate (i.e., 113.64 μM/min), were obtained with respect to those found using OPDA at pH 7.0 (i.e., 54.35 μM/min) (Figure 10B,C). Hence, the enhancement of H_2_O_2_ formation in the acidic region via the reaction of the CaO_2_ nanoshell with H_2_O was confirmed with the higher TMB conversion rates obtained with CuO@CaO_2_ NSs at pH 5.0 (Figure 10C).

The main findings obtained in the POD-like activity runs can be summarized as follows:

(I) POD-like activity of CuO NSs confirmed the ^•^OH radical generation ability of this nanomaterial.
(II) The apparent POD-like activity of CuO@CaO_2_ NSs determined using either externally added or self-supplied H_2_O_2_. The self-POD-like activity of CuO@CaO_2_ NSs determined using self-supplied H_2_O_2_ confirmed the ^•^OH radical generation ability of CuO@CaO_2_ NSs.

The OD-like activity was determined for only CuO NSs since the self-generation of H_2_O_2_ by CuO@CaO_2_ NSs prevented the exact determination of their OD-like activity. The OD-like activity of CuO NSs was studied using two different substrates whose optimum pH values were different in the colorimetric conversions via OD-like activity.^50^ For this reason, OPDA and TMB were selected to work at pH 7.0 and 5.0, respectively. The Michaelis–Menten plots obtained with OPDA at pH 7.0 and TMB at pH 5.0 are given in Figure 10D,E, respectively. The comparison of both plots demonstrated that the maximum substrate consumption rate obtained in acidic pH with TMB via OD-like activity of CuO NSs was considerably higher with respect to that found for neutral pH with OPDA (Figure 10D,E).

CAT-like activity of CuO@CaO_2_ NSs was not determined due to the self-generation of H_2_O_2_ by CaO_2_ nanoshell, which caused an interference for the H_2_O_2_ concentration in the reaction medium. In other words, the resulting H_2_O_2_ concentration in the reaction medium containing CuO@CaO_2_ NSs changes depending upon either the consumption of H_2_O_2_ due to the CAT-like activity of the nanozyme or the production of H_2_O_2_ by the reaction of CaO_2_ nanoshell with H_2_O. Hence it is not possible to predict the decay in the H_2_O_2_ concentration depending on only the CAT-like activity of the nanozyme under these conditions. However, the CAT-like activity of CuO NSs was determined since H_2_O_2_ initially loaded in the reaction medium was smoothly consumed via the CAT-like activity of this nanozyme. The Michaelis–Menten plot for the CAT-like activity of CuO NSs is given in Figure 10F. As seen here, the maximum H_2_O_2_ consumption rate for the CAT-like activity of CuO NSs was determined as 0.122 mM/min. Note that all related Lineweaver–Burk plots for the POD-like, self-POD-like, OD-like, and CAT-like activities of different NSs are given in Figure S10 of the Supporting Information.

### 3.6 Photothermal Conversion Behavior of CuO@CaO_2_@Ce6 NSs

**Figure 11:**
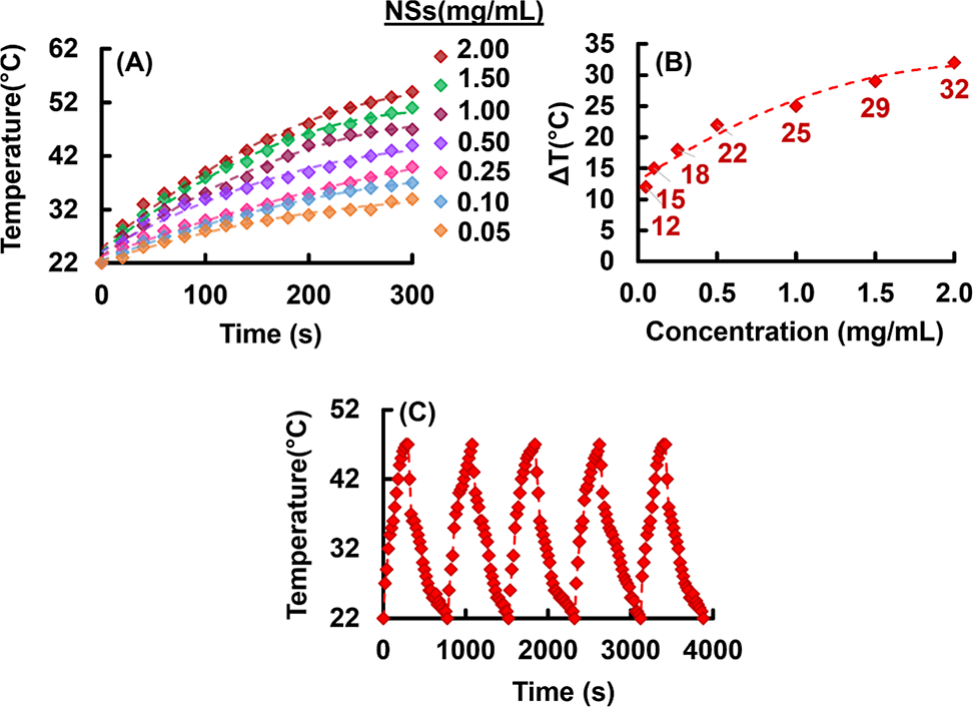
(A) Temperature elevation curves obtained with different concentrations of CuO@CaO_2_@Ce6 NSs under NIR laser (808 nm) irradiation: power density: 1.3 W/cm^2^. (B) Change of total temperature increases in 300 s with the concentration of CuO@CaO_2_@Ce6 NSs. (C) Consecutive heating/cooling curves with CuO@CaO_2_@Ce6 NSs at a concentration of 1.0 mg/mL.

CuO@CaO_2_@Ce6 NSs exhibited a photothermal conversion ability. As seen in Figure 11A,B, the temperature elevations ranging between 15 and 32 °C were obtained in 5 min by changing the concentrations of CuO@CaO_2_@Ce6 NSs in the range of 0.1–2.0 mg/mL under NIR laser irradiation at 808 nm. These plots revealed the suitability of CuO@CaO_2_@Ce6 NSs for photothermal therapy. CuO@CaO_2_@Ce6 NSs exhibited reversible behavior in NIR laser light driven heating and cooling cycles (Figure 11C).

A typical consecutive heating–cooling cycle obtained using CuO@CaO_2_@Ce6 NSs at a concentration of 1.0 mg/mL is given in Figure S11A. The photothermal conversion efficiency of CuO@CaO_2_@Ce6 NSs was calculated as 43% according to the time constant method as described previously.^50^ The Tauc plot sketched for CuO NSs is given in Figure S11B. The extrapolation of the linear part of the Tauc plot provided a low band gap energy value of 1.5 eV for CuO NSs. Tenorite is the mineral name for CuO, which has been described as an inorganic material with p-type semiconductor properties with the band gap energies ranging between 1.2 and 1.9 eV. The band gap energy determined for CuO NSs is consistent with the previously reported band gap range of CuO.^83,84^

### 3.7 Interaction of CuO@CaO_2_@Ce6 NSs with T98G Glioblastoma Cells Using Photothermal, Chemodynamic, and Photodynamic Modalities

In the first group of these runs, the cytotoxicity of CuO NSs was investigated by the determination of the viability of L929 cells that interacted with CuO NSs. The live/dead L929 cell images taken after dual cell staining with AO/PI system, following the interaction with CuO NSs at different concentrations are included in Figure S12A of the Supporting Information. The MTT results exemplifying the viability of L929 cells after interaction with CuO NSs are presented in Figure S12B of the Supporting Information. No significant in vitro cytotoxicity was observed for CuO NSs up to a concentration of 1.5 mg/mL.

**Figure 12:**
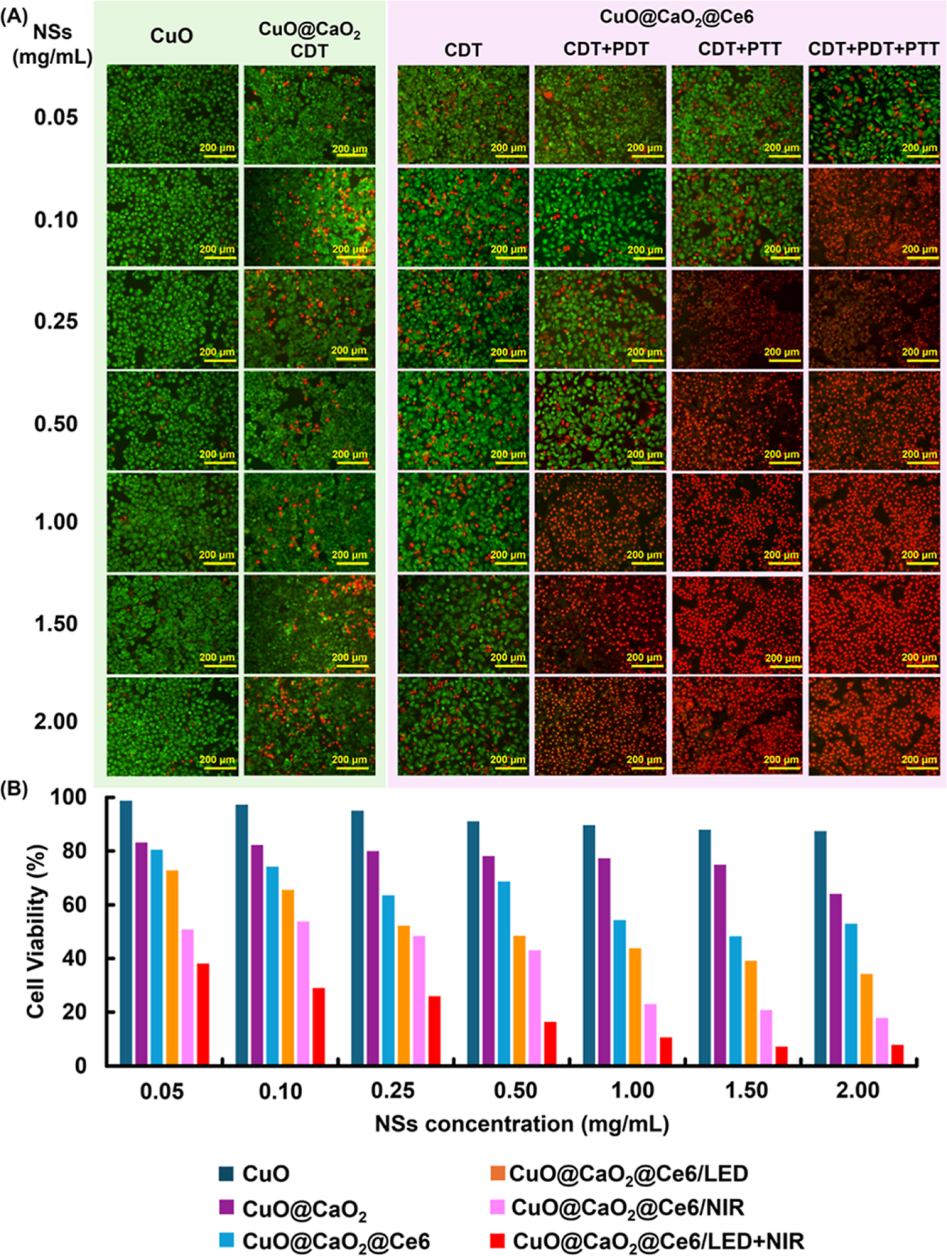
(A) Representative live/dead cell images of T98G cells stained with AO/PI after treatment with CuO, CuO@CaO_2_, and CuO@CaO_2_@Ce6 NSs under different conditions. The images in the first three columns were obtained without using any light irradiation in the presence of CuO, CuO@CaO_2_, and CuO@CaO_2_@Ce6 NSs. PDT effect was applied by LED irradiation for 7 min at different concentrations of CuO@CaO_2_@Ce6 NSs. The PTT effect was applied by NIR laser irradiation for 5 min at different concentrations of CuO@CaO_2_@Ce6 NSs. Simultaneous PDT + PTT effects were applied by LED irradiation for 7 min and NIR laser irradiation for 5 min at different concentrations of CuO@CaO_2_@Ce6 NSs. T98G cell density: 2 × 10^4^ cells/well, scale bar: 200 μm. (B) MTT results for the viability of T98G glioblastoma cells after treatment with CuO, CuO@CaO_2_, and CuO@CaO_2_@Ce6 NSs at different concentrations under LED (650 nm) and NIR laser (808 nm) irradiations. T98G concentration: 2 × 10^4^ cells/well, LED irradiation time: 7 min (0.8 W), NIR irradiation time: 5 min (1.3 W/cm^2^).

Then, T98G cells interacted with CuO@CaO_2_@Ce6 NSs using NIR laser at 808 nm and red LED at 650 nm, respectively. In these runs, CuO and CuO@CaO_2_ NSs were included as control agents without any light irradiation. The live/dead cell images stained with AO/PI after interaction with CuO, CuO@CaO_2_, and CuO@CaO_2_@Ce6 NSs using NIR laser at 808 nm and/or red LED at 650 nm, respectively, are given in Figure 12A. Here, live and dead cells were observed with intracellular green and red fluorescence emissions, respectively. Note that the CDT effect was observed in all cases involving CuO@CaO_2_ and CuO@CaO_2_@Ce6 NSs. This is attributed to the generation of H_2_O_2_ from the reaction between the CaO_2_ nanoshell and water, followed by the production of highly reactive ^•^OH radicals through the Fenton-like activity of NSs. The MTT results for T98G cells after interaction with CuO, CuO@CaO_2_ and CuO@CaO_2_@Ce6 NSs at different concentrations under LED (650 nm) and NIR laser (808 nm) irradiations are presented in Figure 12B.

Live/dead cell images and MTT results revealed that when the PDT effect was applied together with the CDT effect by the generation of ^1^O_2_ and ^•^OH radicals under LED light irradiation at 650 nm, cell deaths higher than 50% were observed with CuO@CaO_2_@Ce6 concentrations equal to or higher than 0.5 mg/mL. This finding indicated that the generation of ^1^O_2_ and ^•^OH radicals due to the PDT and CDT functions of CuO@CaO_2_@Ce6 NSs did not cause a serious cell death with the CuO@CaO_2_@Ce6 concentrations lower than 0.5 mg/mL. However, the PTT effect applied by the NIR laser irradiation at 808 nm, together with CDT, caused cell deaths higher than 50%, with the CuO@CaO_2_@Ce6 concentrations equal to or higher than 0.25 mg/mL. Note that the formation of a higher number of toxic ^•^OH radical generation was demonstrated by increasing the temperature from 25 to 45 °C in the fluorescence spectroscopy runs performed with CuO@CaO_2_ NSs (Figure 8A). In this set, CDT and PDT functions should be more effective by the generation of a higher number of ^•^OH and ^1^O_2_ radicals, respectively, due to the temperature elevation taking place under NIR light irradiation. The cell deaths higher than 60% were achieved with the CuO@CaO_2_@Ce6 NS concentrations starting from 0.05 mg/mL when PDT, CDT, and PTT actions were activated under LED and NIR laser irradiations (Figure 12A,B). The cell death markedly increased with the increasing concentration of CuO@CaO_2_@Ce6 NSs under LED and NIR laser irradiations (Figure 12B).

The application of PDT together with CDT and PTT resulted in cell deaths up to 90% with the CuO@CaO_2_@Ce6 concentration of 1.0 mg/mL. Note that a significant cytotoxicity with L929 cells was not observed for this concentration of CuO@CaO_2_@Ce6 NSs. The cell deaths higher than 90% were achieved with the CuO@CaO_2_@Ce6 concentrations higher than 1.0 mg/mL. However, a noticeable cytotoxicity ca. 16–18% was also observed for L929 cells with the concentrations higher than 1.0 mg/mL (i.e., for 1.5 and 2.0 mg/mL in Figure S12B). The combination of GSH depletion and H_2_O_2_ generation abilities provided more effective ^•^OH radical production in the cell culturing medium. The simultaneous application of PTT also enhanced the GSH depletion and H_2_O_2_ generation abilities, which in turn enhanced the CDT function via ^•^OH radical generation ability due to the temperature elevation. The nanomotor function also likely allowed for a more effective interaction of CuO@CaO_2_@Ce6 NSs with the tumor cells. Hence, seriously high cell deaths were achieved under in vitro conditions by the combination of PDT, PTT, and CDT effects with CuO@CaO_2_@Ce6 NSs.

Cell death observed by AO/PI staining was also confirmed by the TUNEL assay used for analyzing DNA fragmentation as a biomarker of apoptosis in T98G cells. The cell images obtained under inverted microscope with ×100 and ×200 magnifications after the TUNEL assay are given in Figure S13A,B of the Supporting Information, respectively. As seen in the control images obtained with both magnifications in Figure S13A,B, the TUNEL-negative, live cells were identified with beige color. The cells undergoing apoptosis termed as TUNEL positive cells displayed a brown color due to the formation of brown stained nuclei. The concentration of T98G cells undergoing apoptosis was relatively lower in all therapeutic effects when the concentration of CuO@CaO_2_@Ce6 NSs was 0.1 mg/mL. The concentration of marked apoptotic cells seriously increased when only chemodynamic + photodynamic, only chemodynamic + photothermal, and chemodynamic + photodynamic + photothermal effects were applied with the CuO@CaO_2_@Ce6 concentration of 1 mg/mL. The dense dark brown regions found in the images obtained with combinatorial chemodynamic + photodynamic + photothermal effects with the NS concentration of 1 mg/mL provided significant cell death in this mode. These TUNEL assay results are given in Figure S13, which are consistent with the AO/PI staining data in Figure 12.

Cell migration and invasion are the properties controlling the metastatic behavior of cancer cells.^85,86^ The effect of synergistic effects driven by CuO@CaO_2_@Ce6 NSs on the migration of T98G cells was investigated by performing a scratch wound healing assay. The progress of the wound closure by the migrating cells in the scratched area was followed under an inverted microscope for 36 h. The images of the scratched area were taken at 6, 24, and 36 h. The wound closure and cell migration potentials of all groups were determined by making crystal violet staining at 36 h. The inverted optical microcopy images of T98G cells subjected to scratch assay by applying different synergistic effects with CuO@CaO_2_@Ce6 NSs are given in Figure S14A–C of the Supporting Information for control groups not containing CuO@CaO_2_@Ce6 NSs; the scratched areas were subjected to synergistic effects with the CuO@CaO_2_@Ce6 NS concentrations of 0.1 and 0.5 mg/mL, respectively. As expected, no appreciable wound healing was observed in the control group not containing FBS in 36 h (Figure S14A(i)). A noticeable wound closure demonstrating the metastatic characteristics of T98G glioblastoma cells was obtained in the control group when FBS was added into the cell culture medium (Figure S14A(ii)). A marked wound healing ability was also observed when allantoin was included as an agent inducing the wound healing mechanism (Figure S14A(iii)). When the chemodynamic + photodynamic effects were simultaneously applied with the CuO@CaO_2_@Ce6 NS concentration of 0.1 mg/mL, an appreciable wound healing was also observed (Figure S14B(i)). This finding indicated that simultaneous application of chemodynamic + photodynamic effects with the low concentration of CuO@CaO_2_@Ce6 NSs did not result in an appreciable inhibition in cell motility and migration and in turn the wound healing process. However, the application of chemodynamic + photothermal effects and also chemodynamic + photothermal + photodynamic effects with the CuO@CaO_2_@Ce6 NS concentrations of 0.1 and 0.5 mg/mL resulted in almost complete inhibition of the cell proliferation and migration processes [Figure S14B(ii,iii) and all cases in Figure S14C], and no appreciable wound healing was observed. The MTT results given in Figure 12B also demonstrated that higher cell deaths with respect to that obtained with the CuO@CaO_2_@Ce6 NS concentration of 0.1 mg/mL, using combinatorial application of chemodynamic + photodynamic effects, were achieved by either the simultaneous applications of chemodynamic + photothermal effects or chemodynamic + phodynamic + photothermal effects with the CuO@CaO_2_@Ce6 NS concentrations higher than 0.1 mg/mL.

## 4 Conclusions

In this work, CuO@CaO@Ce6 NSs are engineered as a new catalytic nanoreactor for the production and recycling of ROS in TME for the amplification of oxidative stress. CuO@CaO_2_@Ce6 NSs act as a self-propelling nanozyme, which is capable of autonomously navigating in TME to enhance the possibility of interaction with the tumor cells. Low oxygen concentration in TME is addressed as a major limitation of CDT. The CAT-like property of the self-propelling nanozyme allows one to apply hypoxia-tolerant CDT. By considering these properties, CuO@CaO_2_@Ce6 NSs are proposed as a new multifunctional, potential nanoplatform for enhanced cancer therapy.

For this purpose, a new staged shape-templating hydrothermal method was developed for the synthesis of CuO NSs for the first time. The proposed protocol might be also suitable for the production of similar NSs based on different transition metal oxides (i.e., Fe_2_O_3_, Co_3_O_4_, MoO_3_, MnO_2_ etc.) and also noble metals (Ag, Pd, Pt, Ir, etc.). The main properties of CuO NSs produced in this work can be listed below:

(I) CuO NSs acted as a nanozyme with CAT-like, OD-like, and POD-like activities.
(II) Due to CAT-like activity, CuO NSs exhibited a H_2_O_2_-fueled nanomotor behavior allowing for the self-propelled diffusion in the aqueous medium by the generation of O_2_ bubbles via the decomposition of H_2_O_2_. This property should be evaluated as a superiority increasing the interaction possibility of CuO NSs with the tumor cells.
(III) CuO NSs have a Fenton-like activity due to the generation of Cu(I) cations by their GSH depletion ability.
(IV) Excellent photothermal performance was observed with CuO@CaO_2_@Ce6 NSs under NIR laser irradiation with the temperature elevations up to 25 °C using the concentrations lower than 1.0 mg/mL.
(V) By the integration of CaO_2_ onto CuO NSs, a nanozyme with Cu(II)/Cu(I) redox cycling ability was synthesized. This property allowed for the depletion of GSH with higher consumption rates using CuO@CaO_2_ NSs. The Fenton-like activity of CuO NSs was enhanced due to H_2_O_2_ generation by the reaction of CaO_2_ nanoshell on CuO NSs with H_2_O. Hence, the toxic ^•^OH radical production ability of CuO NSs was also elevated.
(VI) It was also demonstrated that the generation rate of ^•^OH radical was elevated by increasing the temperature. This finding should trigger the formation of a synergy with higher GSH depletion ability, higher Fenton-like activity, and in turn more effective CDT due to the temperature elevation originated from photothermal conversion.
(VII) The interaction of CuO@CaO_2_@Ce6 NSs with T98G glioblastoma cells provided cell deaths higher than 90% by the combination of visible light and NIR laser light irradiations.
(VIII) CuO NSs produced in this work can be evaluated as a high performance nanoplatform due to their superior properties listed above and may open a new path for the synthesis of new therapy agents for various tumor cells and also various antibacterial applications.

## Supporting Information Available

The movement of individual spindle-like CuO@CaO_2_ NSs observed with the small oxygen bubbles generated and then bursted around them (MP4)
The trajectory of the self-propelled motion originated from the nanomotor function of CuO@CaO_2_ NSs (MP4)
The convective motion of CuO@CaO_2_ clusters containing large number of NSs observed in the form of gray clouds moving away from the microaggregates (MP4)
Stability of Ce6 on CuO@CaO_2_ NSs; the SEM photographs of CuO, CuO@CaO_2_, and CuO@CaO_2_@Ce6 NSs with high magnification, NS type and magnification: (A) CuO, 74.9 KX, (B) CuO@CaO_2_, 50.0 KX, and (C) CuO@CaO_2_@Ce6, 50.0 KX, variation of Cu(II) adsorption onto poly(GDMA-*co*-MAA) nanospheres with the time, conditions: CuCl_2_·2H_2_O: 200 mg, poly(GDMA-*co*-MAA) nanospheres: 100 mg, DI water: 40 mL, stirring rate: 300 rpm, room temperature, EDX spectroscopy images of CuO NSs, survey XPS of CuO and CuO@CaO_2_@Ce6 NSs, surface atomic compositions of CuO and CuO@CaO_2_@Ce6 NSs determined by XPS, XPS of CuO NSs, (A) Cu 2p scan, (B) O 1s scan, and (C) C 1s scan, the effect of nanomotor function on the interaction of CuO and CuO@CaO_2_ NSs with T98G cells, the inverted microscopy images of (A) nonadherent T98G cell suspension containing no NSs and no H_2_O_2_, (B) nonadherent T98G cell suspension containing only CuO NSs, (C) nonadherent T98G cell suspension containing only CuO@CaO_2_ NSs, (D) nonadherent T98G cell suspension containing CuO NSs and 1 mM H_2_O_2_, and (E) nonadherent T98G cell suspension containing CuO@CaO_2_ NSs and 1 mM H_2_O_2_, cell density: 2 × 10^4^, concentration of NSs: 0.5 mg/mL, interaction time: 10 min, 37 °C, sample UV–vis spectra recorded for GSH depletion using DTNB as the reactive probe with low and high concentrations of CuO and CuO@CaO_2_, (A) CuO NSs (0.1 mg/mL), (B) CuO NSs (2.0 mg/mL), (C) CuO@CaO_2_ NSs (0.1 mg/mL), and (D) CuO@CaO_2_ NSs (2.0 mg/mL), the variation of intracellular GSH content with the concentration of CuO and CuO@CaO_2_ NSs, conditions: cell density: 4 × 10^4^ cells/well, time: 60 min, (A) the variation of equilibrium Ce6 adsorption onto CuO@CaO_2_ NSs with the initial Ce6 concentration, (B) the cumulative release of Ce6 from CuO@CaO_2_@Ce6 NSs in phosphate buffer at pH 5.5 and 7.0. (Figure S9); Lineweaver–Burk plots for (A) POD-like activities of CuO and CuO@CaO_2_ NSs, (B) self-POD-like activity of CuO@CaO_2_ NSs without adding H_2_O_2_ using OPDA as the substrate at pH 7.0, (C) self-POD-like activity of CuO@CaO_2_ NSs without adding H_2_O_2_ using TMB as the substrate at pH 5.0, (D) OD-like activity of CuO NSs using OPDA as the substrate at pH 7.0, (E) OD-like activity of CuO NSs using TMB as the substrate at pH 5.0, and (F) CAT-like activity of CuO NSs, nanozyme concentration: 2.0 mg/mL, temperature: 25 °C, comparison of *K*_m_ and *V*_max_ values of CuO and CuO@CaO_2_ NSs, (A) a typical consecutive heating cooling cycle obtained using CuO@CaO_2_@Ce6 NSs at a concentration of 1.0 mg/mL and the determination of photothermal efficiency (η) of CuO@CaO_2_@Ce6 NSs according to the time constant method (Inset), (B) the Tauc plot sketched for CuO NSs for the determination of band gap energy, the viability of L929 cells with CuO NSs at different concentrations, (A) live/dead L929 cell images taken after dual cell staining with AO/PI system, after treatment with CuO NSs with different concentrations, scale bar: 200 μm, L929 cell density: 2 × 10^4^ cells/well, (B) MTT results exemplifying the viability of L929 cells after treatment with CuO NSs with different concentrations, the control image was obtained without including CuO NSs in the well, representative inverted microscope images of apoptotic T98G cells identified by terminal deoxynucleotidyl TUNEL assay, magnification: panel A: ×100, panel B: ×200, conditions: T98G concentration: 2 × 10^4^ cells/well, LED irradiation time: 7 min (0.8 W) for chemodynamic + photodynamic effects, NIR laser (808 nm) irradiation time: 5 min (1.3 W/cm^2^) for chemodynamic + photothermal effects, LED irradiation time: 7 min (0.8 W) + NIR laser (808 nm) irradiation time: 5 min (1.3 W/cm^2^) for chemodynamic + photodynamic + photothermal effects, concentration of CuO@CaO_2_@Ce6 NSs: 0.1 and 1.0 mg/mL, the inverted optical microcopy images of T98G cells subjected to scratch assay for cell proliferation and migration, the images of control groups prepared with (i) DMEM without FBS, (ii) DMEM with FBS, (iii) DMEM containing allantoin, the images of cells interacted with 0.1 mg/mL of CuO@CaO_2_@Ce6 NSs by applying (i) chemodynamic + photodynamic, (ii) chemodynamic + photothermal, and (iii) chemodynamic + photodynamic + photothermal effects, and the images of cells interacted with 0.5 mg/mL of CuO@CaO_2_@Ce6 NSs by applying (i) chemodynamic + photodynamic, (ii) chemodynamic + photothermal, and (iii) chemodynamic + photodynamic + photothermal effects (Figure S14) (PDF)

## References

[ref1] Tian Q.; Li S.; Tang Z.; Zhang Z.; Du D.; Zhang X.; Niu X.; Lin Y. Nanozyme-Enabled Biomedical Diagnosis: Advances, Trends, and Challenges. Adv. Healthcare Mater. 2024, 2401630 10.1002/adhm.202401630. 39139016

[ref2] Lee J.; Liao H.; Wang Q.; Han J.; Han J. H.; Shin H. E.; Ge M.; Park W.; Li F. Exploration of nanozymes in viral diagnosis and therapy. Exploration 2022, 2, 20210086 10.1002/EXP.20210086. 37324577 PMC10191057

[ref3] You K.; Wang Q.; Osman M. S.; Kim D.; Li Q.; Feng C.; Wang L.; Yang K. Advanced strategies for combinational immunotherapy of cancer based on polymeric nanomedicines. BMEMat 2024, 2, e12067 10.1002/bmm2.12067.

[ref4] He S.-B.; Balasubramanian P.; Hu A.-L.; Zheng X.-Q.; Lin M.-T.; Xiao M.-X.; Peng H.-P.; Deng H.-H.; Chen W. One-pot cascade catalysis at neutral pH driven by CuO tandem nanozyme for ascorbic acid and alkaline phosphatase detection. Sens. Actuators, B 2020, 321, 128511 10.1016/j.snb.2020.128511.

[ref5] Song Y.; Lu S.; Hai J.; Liang K.; Sun S.; Meng G.; Wang B. Nitrogen-Doped Chiral CuO/CoO Nanofibers: An Enhanced Electrochemiluminescence Sensing Strategy for Detection of 3,4-Dihydroxy-Phenylalanine Enantiomers. Anal. Chem. 2021, 93 (33), 11470–11478. 10.1021/acs.analchem.1c01497. 34379390

[ref6] Zhu J.; Cui Q.; Wen W.; Zhang X.; Wang S. Cu/CuO-Graphene Foam with Laccase-like Activity for Identification of Phenolic Compounds and Detection of Epinephrine. Chem. Res. Chin. Univ. 2022, 38 (4), 919–927. 10.1007/s40242-022-2114-x.

[ref7] Wang K.; Liu J.; Wang X.; Liu X.; Hu J.; Li E.; Zhao Y.; Zhao R.; Yang S. Ratiometric fluorescent detection system based on dual-driving catalysis of CuO nanozyme with a classical univariate calibration for the determination of ascorbic acid in serum and fruits. Microchem. J. 2022, 172, 106921 10.1016/j.microc.2021.106921.

[ref8] Hao J.; Feng J.; Sun S.; Cao Z.; Xu W.; Hu L.; Yao W.; Yan Z. Reliable ratiometric colorimetric monitoring of dopamine in practice based on the catalytic signal amplification of nano CeO_2_/CuO modified carboxylated chitosan. Chem. Eng. Sci. 2024, 295, 120193 10.1016/j.ces.2024.120193.

[ref9] Lu M.; Wang Z.; Xie W.; Zhang Z.; Su L.; Chen Z.; Xiong Y. Cu-MOF derived CuO@g-C_3_N_4_ nanozyme for cascade catalytic colorimetric sensing. Anal. Bioanal. Chem. 2023, 415 (24), 6133 10.1007/s00216-023-04892-4. 37581708

[ref10] Zhu W.; Cheng Y.; Wang C.; Lu X. Fabrication of a Tubular CuO/NiO Biomimetic Nanozyme with Synergistically Promoted Peroxidase-like Performance for Isoniazid Sensing. Inorg. Chem. 2022, 61 (41), 16239–16247. 10.1021/acs.inorgchem.2c01896. 36179151

[ref11] Tian L.; Qi J.; Qian K.; Oderinde O.; Liu Q.; Yao C.; Song W.; Wang Y. Copper (II) oxide nanozyme based electrochemical cytosensor for high sensitive detection of circulating tumor cells in breast cancer. J. Electroanal. Chem. 2018, 812, 1–9. 10.1016/j.jelechem.2017.12.012.

[ref12] Zhuang Z.; Zhang C.; Yu Z.; Liu W.; Zhong Y.; Zhang J.; Xu Z. Turn-on colorimetric detection of hydroquinone based on Au/CuO nanocomposite nanozyme. Mikrochim. Acta 2022, 189 (8), 293 10.1007/s00604-022-05384-5. 35881205

[ref13] Ali S.; Sikdar S.; Basak S.; Das D.; Roy D.; Salman Haydar M.; Kumar Dakua V.; Adhikary P.; Mandal P.; Nath Roy M. Synthesis of β-cyclodextrin grafted rhombohedral-CuO antioxidant nanozyme for detection of dopamine and hexavalent chromium through off–on strategy of peroxidase mimicking activity. Microchem. J. 2022, 179, 107514 10.1016/j.microc.2022.107514.

[ref14] Karim M. N.; Singh M.; Weerathunge P.; Bian P.; Zheng R.; Dekiwadia C.; Ahmed T.; Walia S.; Della Gaspera E.; Singh S.; Ramanathan R.; Bansal V. Visible-Light-Triggered Reactive-Oxygen-Species-Mediated Antibacterial Activity of Peroxidase-Mimic CuO Nanorods. ACS Appl. Nano Mater. 2018, 1 (4), 1694–1704. 10.1021/acsanm.8b00153.

[ref15] Zhuang Q.-Q.; Deng Q.; He S.-B.; Chen Q.-Q.; Peng H.-P.; Deng H.-H.; Xia X.-H.; Chen W. Bifunctional cupric oxide nanoparticle-catalyzed self-cascade oxidation reactions of ascorbic acid for bacterial killing and wound disinfection. Composites, Part B 2021, 222, 109074 10.1016/j.compositesb.2021.109074.

[ref16] Zhuang Q.-Q.; Zhang Z.-S.; Zheng T.-J.; Lu L.-Y.; Lin M.-T.; Yang J.-L.; Deng H.-H.; Xu Y.-Y.; Chen W. Alkaline phosphatase-activated prodrug system based on a bifunctional CuO NP tandem nanoenzyme for on-demand bacterial inactivation and wound disinfection. J. Nanobiotechnol. 2024, 22 (1), 485 10.1186/s12951-024-02751-7. PMC11320994 39138462

[ref17] Esmaeili Govarchin Ghaleh H.; Zarei L.; Mansori Motlagh B.; Jabbari N. Using CuO nanoparticles and hyperthermia in radiotherapy of MCF-7 cell line: synergistic effect in cancer therapy. Artif. Cells, Nanomed., Biotechnol. 2019, 47 (1), 1396–1403. 10.1080/21691401.2019.1600529. 30964344

[ref18] Singh S.; Pal K. Folic-acid adorned alginate-polydopamine modified paclitaxel/Zn-CuO nanocomplex for pH triggered drug release and synergistic antitumor efficacy. Int. J. Biol. Macromol. 2023, 234, 123602 10.1016/j.ijbiomac.2023.123602. 36773860

[ref19] Zhou M.; Tian B.; Bu Y.; Wu Z.; Yu J.; Wang S.; Sun X.; Zhu X.; Zhou H. Enhanced pH-Responsive Chemo/Chemodynamic Synergistic Cancer Therapy Based on In Situ Cu^2+^ Di-Chelation. ACS Appl. Bio Mater. 2023, 6 (8), 3221–3231. 10.1021/acsabm.3c00323. 37428493

[ref20] Singh S.; Pal K. Folate functionalized multifunctional CuO@PHBV@PDA nanoplatform for pH-Responsive dual drug delivery and ROS-driven apoptosis in breast cancer. J. Drug Delivery Sci. Technol. 2024, 96, 105669 10.1016/j.jddst.2024.105669.

[ref21] Wang W.; Zheng T.; Zhang M.; Zhang Q.; Wu F.; Liu Y.; Zhang L.; Zhang J.; Wang M.; Sun Y. Tumor-targeting multi-shelled hollow nanospheres as drug loading platforms for imaging-guided combinational cancer therapy. Biomater. Sci. 2020, 8 (6), 1748–1758. 10.1039/C9BM01881F. 32002530

[ref22] Gao X.; Feng J.; Lv K.; Zhou Y.; Zhang R.; Song S.; Zhang H.; Wang D. Engineering CeO_2_/CuO heterostructure anchored on upconversion nanoparticles with boosting ROS generation-primed apoptosis-ferroptosis for cancer dynamic therapy. Nano Res. 2023, 16 (4), 5322–5334. 10.1007/s12274-022-5223-4.

[ref23] Singh S.; Pal K. Exploration of polydopamine capped bimetallic oxide (CuO-NiO) nanoparticles inspired by mussels for enhanced and targeted paclitaxel delivery for synergistic breast cancer therapy. Appl. Surf. Sci. 2023, 626, 157266 10.1016/j.apsusc.2023.157266.

[ref24] Lin J.; Huang C.; Wang P.; He Y.; Luo Q.; Liu X.; Li Y. Tumor-Microenvironment-Responsive Cerium-Enriched Copper Nanozyme with O_2_ Supply and Oxidative Stress Amplification for In Situ Disulfiram Chemotherapy and Chemodynamic Therapy Intensification. Adv. Healthcare Mater. 2024, 13 (11), 2303955 10.1002/adhm.202303955. 38271271

[ref25] Jiang F.; Ding B.; Liang S.; Zhao Y.; Cheng Z.; Xing B.; Ma P. a.; Lin J. Intelligent MoS2–CuO heterostructures with multiplexed imaging and remarkably enhanced antitumor efficacy via synergetic photothermal therapy/chemodynamic therapy/immunotherapy. Biomater 2021, 268, 120545 10.1016/j.biomaterials.2020.120545. 33253965

[ref26] Xu X.; Li H.; Tong B.; Zhang W.; Wang X.; Wang Y.; Tian G.; Xu Z.; Zhang G. Biomimetic Nano-Regulator that Induces Cuproptosis and Lactate-Depletion Mediated ROS Storm for Metalloimmunotherapy of Clear Cell Renal Cell Carcinoma. Adv. Healthcare Mater. 2024, 13, 2400204 10.1002/adhm.202400204. 38855966

[ref27] Wang D.; Liao Y.; Yan H.; Zhu S.; Liu Y.; Li J.; Wang X.; Guo X.; Gu Z.; Sun B. In Situ Formed Z-Scheme Graphdiyne Heterojunction Realizes NIR-Photocatalytic Oxygen Evolution and Selective Radiosensitization for Hypoxic Tumors. ACS Nano 2022, 16 (12), 21186–21198. 10.1021/acsnano.2c09169. 36445074

[ref28] Wang J.; Ye J.; Lv W.; Liu S.; Zhang Z.; Xu J.; Xu M.; Zhao C.; Yang P.; Fu Y. Biomimetic Nanoarchitectonics of Hollow Mesoporous Copper Oxide-Based Nanozymes with Cascade Catalytic Reaction for Near Infrared-II Reinforced Photothermal-Catalytic Therapy. ACS Appl. Mater. Interfaces 2022, 14 (36), 40645–40658. 10.1021/acsami.2c11634. 36040363

[ref29] Zhang G.; Xie W.; Xu Z.; Si Y.; Li Q.; Qi X.; Gan Y.; Wu Z.; Tian G. CuO dot-decorated Cu@Gd_2_O_3_ core–shell hierarchical structure for Cu(i) self-supplying chemodynamic therapy in combination with MRI-guided photothermal synergistic therapy. Mater. Horiz. 2021, 8 (3), 1017–1028. 10.1039/D0MH01685C. 34821332

[ref30] Bai J.; Zhang X.; Zhao Z.; Sun S.; Cheng W.; Yu H.; Chang X.; Wang B. CuO Nanozymes Catalyze Cysteine and Glutathione Depletion Induced Ferroptosis and Cuproptosis for Synergistic Tumor Therapy. Small 2024, 20 (40), 2400326 10.1002/smll.202400326. 38813723

[ref31] Jia S.; Ke S.; Tu L.; Chen S.; Luo B.; Xiong Y.; Li Y.; Wang P.; Ye S. Glutathione/pH-responsive copper-based nanoplatform for amplified chemodynamic therapy through synergistic cycling regeneration of reactive oxygen species and dual glutathione depletion. J. Colloid Interface Sci. 2023, 652, 329–340. 10.1016/j.jcis.2023.08.043. 37597414

[ref32] Pang Y.; Lv J.; He C.; Ju C.; Lin Y.; Zhang C.; Li M. Covalent organic frameworks-derived carbon nanospheres based nanoplatform for tumor specific synergistic therapy via oxidative stress amplification and calcium overload. J. Colloid Interface Sci. 2024, 661, 908–922. 10.1016/j.jcis.2024.01.217. 38330663

[ref33] Zhang F.; Xin C.; Dai Z.; Hu H.; An Q.; Wang F.; Hu Z.; Sun Y.; Tian L.; Zheng X. Oncocyte Membrane-Camouflaged Multi-Stimuli-Responsive Nanohybrids for Synergistic Amplification of Tumor Oxidative Stresses and Photothermal Enhanced Cancer Therapy. ACS Appl. Mater. Interfaces 2022, 14 (36), 40633–40644. 10.1021/acsami.2c11200. 36052606

[ref34] Liu Y.; Chi S.; Cao Y.; Liu Z. Glutathione-Responsive Biodegradable Core–Shell Nanoparticles That Self-Generate H_2_O_2_ and Deliver Doxorubicin for Chemo–Chemodynamic Therapy. ACS Appl. Nano Mater. 2022, 5 (2), 2592–2602. 10.1021/acsanm.1c04277.

[ref35] Jiang Y.; Xu C.; Li Y.; Wang H.; Liu L.; Ye Y.; Gao J.; Tian H.; Peng F.; Tu Y.; Li Y. Bottle Nanomotors Amplify Tumor Oxidative Stress for Enhanced Calcium Overload/Chemodynamic Therapy. Small 2024, 20, 2404402 10.1002/smll.202404402. 38963075

[ref36] Bai Z.; Huang J.; Lu H.; Wang N.; Li H.; Zhu Y. Based on polydopamine-coated metal organic framework multifunctional nanoplatform for enhanced photothermal/sonodynamicand treatment combined with checkpoint blockade therapy. Int. J. Biol. Macromol. 2024, 269, 132207 10.1016/j.ijbiomac.2024.132207. 38723823

[ref37] Zhang L.; Lu H.; Tang Y.; Lu X.; Zhang Z.; Zhang Y.; Liu Y.; Wang C. Calcium-peroxide-mediated cascades of oxygen production and glutathione consumption induced efficient photodynamic and photothermal synergistic therapy. J. Mater. Chem. B 2023, 11 (13), 2937–2945. 10.1039/D2TB02776C. 36912360

[ref38] Chen M.; Zhao S.; Zhu J.; Feng E.; Lv F.; Chen W.; Lv S.; Wu Y.; Peng X.; Song F. Open-Source and Reduced-Expenditure Nanosystem with ROS Self-Amplification and Glutathione Depletion for Simultaneous Augmented Chemodynamic/Photodynamic Therapy. ACS Appl. Mater. Interfaces 2022, 14 (18), 20682–20692. 10.1021/acsami.2c01782. 35500204

[ref39] Huang C.; Lin B.; Chen C.; Wang H.; Lin X.; Liu J.; Ren Q.; Tao J.; Zhao P.; Xu Y. Synergistic Reinforcing of Immunogenic Cell Death and Transforming Tumor-Associated Macrophages Via a Multifunctional Cascade Bioreactor for Optimizing Cancer Immunotherapy. Adv. Mater. 2022, 34 (51), 2207593 10.1002/adma.202207593. 36245299

[ref40] Pang K.; Pidamaimaiti G.; Zhu Y.; Sun D.; Yu B.; Duanmu Z.; Wang F.; Wei X. A phase change material packaged multifunctional nanoplatform integrating hydrogen peroxide self-supply and photothermal response for boosting synergistic chemodynamic and photothermal therapy. New J. Chem. 2023, 47 (33), 15561–15568. 10.1039/D3NJ02341A.

[ref41] Yu H.; Xu G.; Wen C.; Yu B.; Jin Y.; Yin X.-B. Multi-level Reactive Oxygen Species Amplifier to Enhance Photo-/Chemo-Dynamic/Ca^2+^ Overload Synergistic Therapy. ACS Appl. Mater. Interfaces 2024, 16 (15), 18459–18473. 10.1021/acsami.4c00109. 38578815

[ref42] Saraçoǧlu B.; Uğuzdoğan E.; Gölgelioğlu C. ¸.; Tuncel A. Synthesis of Monodisperse Glycerol Dimethacrylate-Based Microgel Particles by Precipitation Polymerization. Ind. Eng. Chem. Res. 2009, 48 (10), 4844–4851. 10.1021/ie801572w.

[ref43] Shi H.; Ban C.; Dai C.; Li C.; Zhou X.; Xia R.; Qian J.; Cao M. Glutathione-depletion reinforced enzyme catalytic activity for photothermal assisted bacterial killing by hollow mesoporous CuO. J. Mater. Chem. B 2022, 10 (43), 8883–8893. 10.1039/D2TB01621D. 36259979

[ref44] Chen Y.-C.; Liu Y.-J.; Lee C.-L.; Pham K.-Y.; Manoharan D.; Thangudu S.; Su C.-H.; Yeh C.-S. Engineering H_2_O_2_ and O_2_ Self-Supplying Nanoreactor to Conduct Synergistic Chemiexcited Photodynamic and Calcium-Overloaded Therapy in Orthotopic Hepatic Tumors. Adv. Healthcare Mater. 2022, 11 (20), 2201613 10.1002/adhm.202201613. 35879269

[ref45] Süngü Akdogan C. ¸. Z.; Akbay Çetin E.; Onur M. A.; Önel S.; Tuncel A. In Vitro Synergistic Photodynamic, Photothermal, Chemodynamic, and Starvation Therapy Performance of Chlorin e6 Immobilized, Polydopamine-Coated Hollow, Porous Ceria-Based, Hypoxia-Tolerant Nanozymes Carrying a Cascade System. ACS Appl. Bio Mater. 2024, 7 (5), 2781–2793. 10.1021/acsabm.3c01181. PMC11110068 38380497

[ref46] Chen T.; Chu Q.; Li M.; Han G.; Li X. Fe_3_O_4_@Pt nanoparticles to enable combinational electrodynamic/chemodynamic therapy. J. Nanobiotechnol. 2021, 19 (1), 206 10.1186/s12951-021-00957-7. PMC8272323 34246260

[ref47] Chen N.; Li Y.; Li H.; Wang Y.; Zeng Y.; Zhang M.; Pan Z.; Chen Z.; Liang W.; Huang J.; Zhang K.; Liu X.; He Y. Multifunctional CuFe_2_O_4_@HA as a GSH-depleting nanoplatform for targeted photothermal/enhanced-chemodynamic synergistic therapy. Colloids Surf., B 2023, 229, 113445 10.1016/j.colsurfb.2023.113445. 37441838

[ref48] Chen T.; Chu Q.; Li M.; Han G.; Li X. Fe 3 O 4@ Pt nanoparticles to enable combinational electrodynamic/chemodynamic therapy. J. Nanobiotechnol. 2021, 19, 1–13. 10.1186/s12951-021-00957-7. PMC8272323 34246260

[ref49] Özcan S.; Süngü Akdoğan C. ¸. Z.; Polat M.; Kip C. ¸.; Tuncel A. A new multimodal magnetic nanozyme and a reusable peroxymonosulfate oxidation catalyst: Manganese oxide coated-monodisperse-porous and magnetic core-shell microspheres. Chemosphere 2023, 341, 140034 10.1016/j.chemosphere.2023.140034. 37659514

[ref50] Sungu Akdogan C. Z.; Gokcal B.; Polat M.; Hamaloglu K. O.; Kip C.; Tuncel A. Porous, Oxygen Vacancy Enhanced CeO_2–x_ Microspheres with Efficient Enzyme-Mimetic and Photothermal Properties. ACS Sustain. Chem. Eng. 2022, 10 (29), 9492–9505. 10.1021/acssuschemeng.2c01981.

[ref51] Erfen S. ¸.; Akbay Çetin E. Therapeutic and preventive effects of piperine and its combination with curcumin as a bioenhancer against aluminum-induced damage in the astrocyte cells. Neurotox. Res. 2022, 40, 2027–2045. 10.1007/s12640-022-00600-9. 36342584

[ref52] Çetin E. A.; Babayiğit E. H.; Özdemir A. Y.; Erfen S. ¸.; Onur M. A. Investigation of UV-treated mesenchymal stem cells in an in vitro wound model. In Vitro Cell. Dev. Biol.: Anim. 2023, 59, 331–345. 10.1007/s11626-023-00772-4. 37296290

[ref53] Cao X.; Yu F.; Li L.; Yao Z.; Xie Y. Copper nanorod junctions templated by a novel polymer–surfactant aggregate. J. Cryst. Growth 2003, 254, 164–168. 10.1016/S0022-0248(03)01115-1.

[ref54] Oaki Y.; Muramatsu R.; Imai H. Polymer-mediated dendritic growth of a transition metal salt crystal as a template for morphogenesis. Polym. J. 2015, 47, 183–189. 10.1038/pj.2014.113.

[ref55] Siddiqui H.; Qureshi M. S.; Haque F. Z. Biosynthesis of Flower-Shaped CuO Nanostructures and Their Photocatalytic and Antibacterial Activities. Nano-Micro Lett. 2020, 12 (1), 29 10.1007/s40820-019-0357-y. PMC7770900 34138069

[ref56] Sahadat Hossain M.; Ahmed S. Sustainable synthesis of nano CuO from electronic waste (E-waste) cable: Evaluation of crystallite size via Scherrer equation, Williamson-Hall plot, Halder- Wagner model, Monshi-Scherrer model, size-strain plot. Results Eng. 2023, 20, 101630 10.1016/j.rineng.2023.101630.

[ref57] Zhang K.; Wu J.; Zhao X.; Qin J.; Xue Y.; Zheng W.; Wang L.; Wang H.; Shen H.; Niu T.; Luo Y.; Tang R.; Wang B. Prussian Blue/Calcium Peroxide Nanocomposites-Mediated Tumor Cell Iron Mineralization for Treatment of Experimental Lung Adenocarcinoma. ACS Nano 2021, 15 (12), 19838–19852. 10.1021/acsnano.1c07308. 34851083

[ref58] Qiu Q.; Li J.; Ren H.; Zhang J.; Liu G.; Yang R.; Sun B.; Zhang C.; Zhang Y. Zinc Coordination Lipid Nanoparticles Co-Delivering Calcium Peroxide and Chelating STING agonist for Enhanced Cancer Metalloimmunotherapy. Small 2024, 20, 2402308 10.1002/smll.202402308. 39114869

[ref59] Vinothkumar V.; Prasad G. V.; Chen S.-M.; Sangili A.; Jang S.-J.; Lim H. C.; Kim T. H. One-step synthesis of calcium-doped copper oxide nanoparticles as an efficient bifunctional electrocatalyst for sensor and supercapacitor applications. J. Energy Storage 2023, 59, 106415 10.1016/j.est.2022.106415.

[ref60] Sun S.; Chen Q.; Tang Z.; Liu C.; Li Z.; Wu A.; Lin H. Tumor Microenvironment Stimuli-Responsive Fluorescence Imaging and Synergistic Cancer Therapy by Carbon-Dot–Cu^2+^ Nanoassemblies. Angew. Chem., Int. Ed. 2020, 59 (47), 21041–21048. 10.1002/anie.202007786. 32914924

[ref61] Dan J.; Rao P.; Wang Q.; Dong L.; Chu W.; Zhang M.; He Z.; Gao N.; Deng J.; Chen J. MgO-supported CuO with encapsulated structure for enhanced peroxymonosulfate activation to remove thiamphenicol. Sep. Purif. Technol. 2022, 280, 119782 10.1016/j.seppur.2021.119782.

[ref62] He Y.; Zhang J.; Zhou H.; Yao G.; Lai B. Synergistic multiple active species for the degradation of sulfamethoxazole by peroxymonosulfate in the presence of CuO@ FeO_x_@ Fe^0^. Chem. Eng. Sci. 2020, 380, 122568 10.1016/j.cej.2019.122568.

[ref63] Liu J.; Liu M.; Chen S.; Wang B.; Chen J.; Yang D.-P.; Zhang S.; Du W. Conversion of Au(III)-polluted waste eggshell into functional CaO/Au nanocatalyst for biodiesel production. Green Energy Environ. 2022, 7 (2), 352–359. 10.1016/j.gee.2020.07.019.

[ref64] Wang Z.; Zhang Y.; Tan Z.; Li Q. A wet process for oxidation-absorption of nitric oxide by persulfate/calcium peroxide. Chem. Eng. Sci. 2018, 350, 767–775. 10.1016/j.cej.2018.05.145.

[ref65] Kawrani S.; Boulos M.; Bekheet M. F.; Viter R.; Nada A. A.; Riedel W.; Roualdes S.; Cornu D.; Bechelany M. Segregation of copper oxide on calcium copper titanate surface induced by Graphene Oxide for Water splitting applications. Appl. Surf. Sci. 2020, 516, 146051 10.1016/j.apsusc.2020.146051.

[ref66] Ren G.; Wang Z.; Tian Y.; Li J.; Ma Y.; Zhou L.; Zhang C.; Guo L.; Diao H.; Li L.; Lu L.; Ma S.; Wu Z.; Yan L.; Liu W. Targeted chemo-photodynamic therapy toward esophageal cancer by GSH-sensitive theranostic nanoplatform. Biomed. Pharmacother. 2022, 153, 113506 10.1016/j.biopha.2022.113506. 36076595

[ref67] Zubair U.; Amici J.; Crespiera S. M.; Francia C.; Bodoardo S. Rational design of porous carbon matrices to enable efficient lithiated silicon sulfur full cell. Carbon 2019, 145, 100–111. 10.1016/j.carbon.2019.01.005.

[ref68] Zhu Y.; Niu X.; Ding C.; Lin Y.; Fang W.; Yan L.; Cheng J.; Zou J.; Tian Y.; Huang W.; Huang W.; Pan Y.; Wu T.; Chen X.; Kang D. Carrier-Free Self-Assembly Nano-Sonosensitizers for Sonodynamic-Amplified Cuproptosis-Ferroptosis in Glioblastoma Therapy. Adv. Sci. 2024, 11 (23), 2402516 10.1002/advs.202402516. PMC11187904 38582500

[ref69] Lazar P.; Mach R.; Otyepka M. Spectroscopic Fingerprints of Graphitic, Pyrrolic, Pyridinic, and Chemisorbed Nitrogen in N-Doped Graphene. J. Phys. Chem. C 2019, 123 (16), 10695–10702. 10.1021/acs.jpcc.9b02163.

[ref70] Wan M.; Chen H.; Wang Q.; Niu Q.; Xu P.; Yu Y.; Zhu T.; Mao C.; Shen J. Bio-inspired nitric-oxide-driven nanomotor. Nat. Commun. 2019, 10, 966 10.1038/s41467-019-08670-8. 30814497 PMC6393443

[ref71] Chen L.; Yuan H.; Chen S.; Zheng C.; Wu X.; Li Z.; Liang C.; Dai P.; Wang Q.; Ma X.; et al. Cost-effective, high-yield production of biotemplated catalytic tubular micromotors as self-propelled microcleaners for water treatment. ACS Appl. Mater. Interfaces 2021, 13, 31226–31235. 10.1021/acsami.1c03595. 34176260

[ref72] Zhang W.; Xiang Y.; Guo Q.; Wang X.; Zhang L.; Guo J.; Cong R.; Yu W.; Liang X.-J.; Zhang J.; et al. Multi-phoretic nanomotor with consistent motion direction for enhanced cancer therapy. Acta Biomater. 2024, 191, 352–368. 10.1016/j.actbio.2024.11.037. 39586348

[ref73] Liu Z.; Li T.; Li N.; Wang Y.; Chen L.; Tang X.; Wan M.; Mao C. GSH-induced chemotaxis nanomotors for cancer treatment by ferroptosis strategy. Sci. China:Chem. 2022, 65, 989–1002. 10.1007/s11426-021-1208-6.

[ref74] Tang Z.; Jiang S.; Tang W.; He Q.; Wei H.; Jin C.; Wang S.; Zhang H. H_2_O_2_ Self-Supplying and GSH-Depleting Nanocatalyst for Copper Metabolism-Based Synergistic Chemodynamic Therapy and Chemotherapy. Mol. Pharm. 2023, 20 (3), 1717–1728. 10.1021/acs.molpharmaceut.2c00937. 36809003

[ref75] Huang J.; Deng G.; Wang S.; Zhao T.; Chen Q.; Yang Y.; Yang Y.; Zhang J.; Nan Y.; Liu Z.; Cao K.; Huang Q.; Ai K. A NIR-II Photoactivatable “ROS Bomb” with High-Density Cu_2_O-Supported MoS_2_ Nanoflowers for Anticancer Therapy. Adv.Sci. 2023, 10 (24), 2302208 10.1002/advs.202302208. PMC10460899 37340606

[ref76] Ma B.; Wang S.; Liu F.; Zhang S.; Duan J.; Li Z.; Kong Y.; Sang Y.; Liu H.; Bu W.; Li L. Self-Assembled Copper–Amino Acid Nanoparticles for in Situ Glutathione “AND” H_2_O_2_ Sequentially Triggered Chemodynamic Therapy. J. Am. Chem. Soc. 2019, 141 (2), 849–857. 10.1021/jacs.8b08714. 30541274

[ref77] Zhang W.-X.; Hao Y.-N.; Gao Y.-R.; Shu Y.; Wang J.-H. Mutual Benefit between Cu(II) and Polydopamine for Improving Photothermal–Chemodynamic Therapy. ACS Appl. Mater. Interfaces 2021, 13 (32), 38127–38137. 10.1021/acsami.1c12199. 34347422

[ref78] Bokare A. D.; Choi W. Review of iron-free Fenton-like systems for activating H_2_O_2_ in advanced oxidation processes. J. Hazard. Mater. 2014, 275, 121–135. 10.1016/j.jhazmat.2014.04.054. 24857896

[ref79] Northup A.; Cassidy D. Calcium peroxide (CaO_2_) for use in modified Fenton chemistry. J. Hazard. Mater. 2008, 152, 1164–1170. 10.1016/j.jhazmat.2007.07.096. 17804164

[ref80] Jia D.; Zou Y.; Zhang Y.; Xu H.; Yang W.; Zheng X.; Zhang Y.; Yu Q. A self-supplied hydrogen peroxide and nitric oxide-generating nanoplatform enhances the efficacy of chemodynamic therapy for biofilm eradication. J. Colloid Interface Sci. 2025, 678, 20–29. 10.1016/j.jcis.2024.08.148. 39178688

[ref81] Niu B.; Liao K.; Zhou Y.; Wen T.; Quan G.; Pan X.; Wu C. Application of glutathione depletion in cancer therapy: Enhanced ROS-based therapy, ferroptosis, and chemotherapy. Biomater 2021, 277, 121110 10.1016/j.biomaterials.2021.121110. 34482088

[ref82] Tomioka D.; Fujita S.; Matsusaki M. Controlled Release of Oxygen from Calcium Peroxide in a Weak Acidic Condition by Stabilized Amorphous Calcium Carbonate Nanocoating for Biomedical Applications. ACS Omega 2024, 9 (5), 5903–5910. 10.1021/acsomega.3c09406. 38343991 PMC10851353

[ref83] Dhineshbabu N. R.; Rajendran V.; Nithyavathy N.; Vetumperumal R. Study of structural and optical properties of cupric oxide nanoparticles. Appl. Nanosci. 2016, 6 (6), 933–939. 10.1007/s13204-015-0499-2.

[ref84] Althamthami M.; Temam H. B.; Temam E. G.; Rahmane S.; Gasmi B.; Hasan G. G. Impact of surface topography and hydrophobicity in varied precursor concentrations of tenorite (CuO) films: a study of film properties and photocatalytic efficiency. Sci. Rep. 2024, 14 (1), 7928 10.1038/s41598-024-58744-x. 38575755 PMC10995127

[ref85] Guo B.; Huang Z.; Shi Q.; Middha E.; Xu S.; Li L.; Wu M.; Jiang J.; Hu Q.; Fu Z.; et al. Organic small molecule based photothermal agents with molecular rotors for malignant breast cancer therapy. Adv. Funct. Mater. 2020, 30, 1907093 10.1002/adfm.201907093.

[ref86] Xu F.; Li M.; Que Z.; Su M.; Yao W.; Zhang Y.; Luo B.; Li Y.; Zhang Z.; Tian J. Combined chemo-immuno-photothermal therapy based on ursolic acid/astragaloside IV-loaded hyaluronic acid-modified polydopamine nanomedicine inhibiting the growth and metastasis of non-small cell lung cancer. J. Mater. Chem. B 2023, 11, 3453–3472. 10.1039/D2TB02328H. 37009696

