# Supplementary material for: Copper(II) Oxide Spindle-like Nanomotors Decorated with Calcium Peroxide Nanoshell as a New Nanozyme with Photothermal and Chemodynamic Functions Providing ROS Self-Amplification, Glutathione Depletion, and Cu(I)/Cu(II) Recycling
Source: ACS Appl Mater Interfaces. 2024 Dec 25;17(1):632–49. doi: 10.1021/acsami.4c17852 (PMC11783533; doi:10.1021/acsami.4c17852)
Supplement: Supplementary file 4 — am4c17852_si_004.pdf [file am4c17852_si_004.pdf]

Supporting Information

Copper (II) oxide spindle-like nanomotors decorated with calcium peroxide nanoshell as a new nanozyme with photothermal and chemodynamic functions providing ROS self-amplification, glutathione depletion and Cu(I)/Cu(II) recycling

Çağıl Zeynep Süngü Akdoğan[1,2], Esin Akbay Çetin[3], Mehmet Ali Onur[1,3],

Selis Önel[1,4], Ali Tuncel[1,4,]*

1 Hacettepe University, Bioengineering Division, Ankara 06800, Turkey

2 Hacettepe University, Graduate School of Science & Engineering, Ankara 06800, Turkey

3 Hacettepe University, Department of Biology, Ankara 06800, Turkey

4 Hacettepe University, Chemical Engineering Department, Ankara 06800, Turkey

* Corresponding author. Email: atuncel@hacettepe.edu.tr

Number of pages: 15

Number of figures: 14

Number of tables: 2

Movie S1: The movement of individual spindle-like CuO@CaO_2 nanospindles (NSs) was observed with the small oxygen bubbles generated and then bursted around them.

Movie S2: The trajectory of the self-propelled motion originated from the nanomotor function of CuO@CaO_2 NSs.

Movie S3: The convective motion of CuO@CaO_2 clusters containing large number of NSs observed in the form of grey clouds moving away from the microaggregates.

S1. Stability of Ce6 on $CuO@CaO_2$ NSs

Briefly, $CuO@CaO_2$ NSs (5 mg) were added to Ce6-methanol solutions (1 mL) containing Ce6 at different concentrations ranging between 0.1- 1.0 mg/mL. The dispersion was stirred for 30 min at room temperature in the dark. $CuO@CaO_2$ NSs were precipitated by centrifugation at 5000 rpm for 10 min. The absorbance of supernatant was measured at 400 nm in a UV−Vis spectrophotometer. The equilibrium Ce6 adsorption (Q_{Ce6}) onto $CuO@CaO_2$ NSs was calculated according to Eqn (1).

After loading, the release of Ce6 from $CuO@CaO_2$ NSs was investigated using PBS buffers in the pH range of 5.5-7.4. Typically, $CuO@CaO_2@Ce6$ NSs (5 mg) were dispersed in PBS buffer (1 mL) at a certain pH. The medium was shaken at 120 cpm at 37°C for 72 h. The samples were withdrawn from the release medium at prescribed times. $CuO@CaO_2@Ce6$ NSs were precipitated by centrifugation and the absorbance of the supernatant was measured at 400 nm in a UV-Vis spectrophotometer.

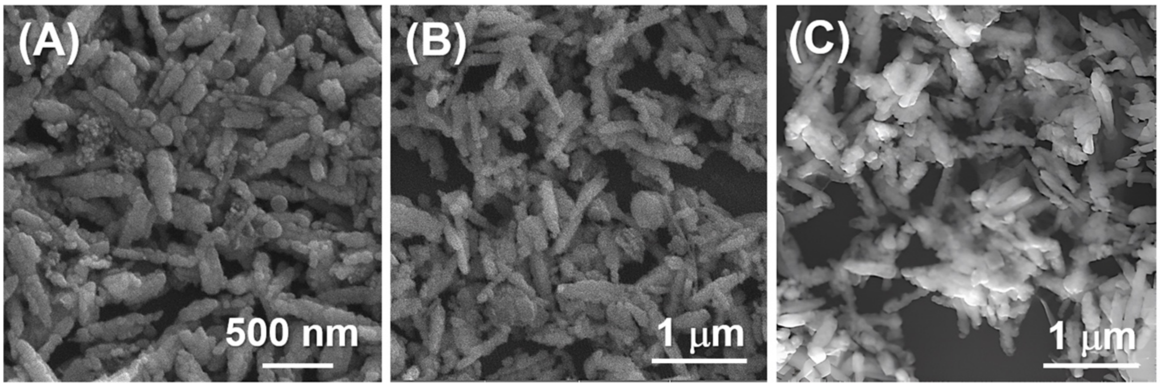

Figure S1. The SEM photographs of CuO, $CuO@CaO_2$ and $CuO@CaO_2@Ce6$ NSs with high magnification**.** Nanospindle (NS) type and magnification: (A) CuO, 74.9 KX, (B) $CuO@CaO_2$, 50.0 KX, (C) $CuO@CaO_2@Ce6$, 50.0 KX.

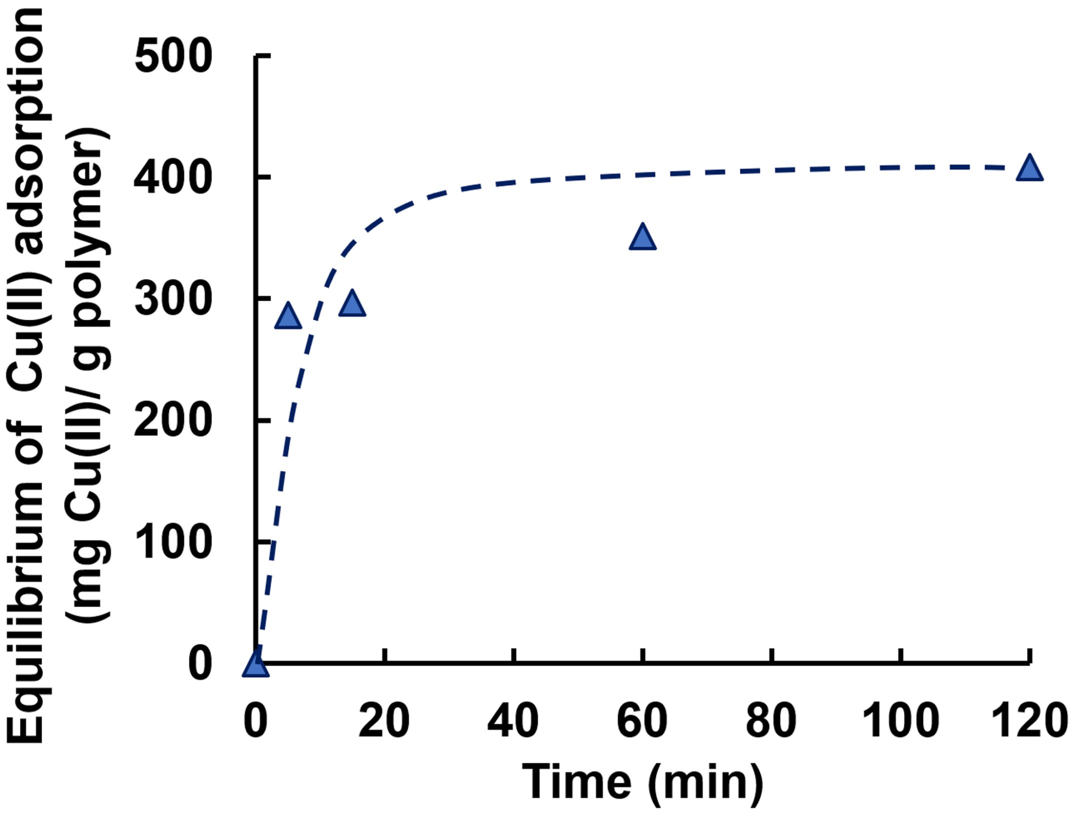

Figure S2. The variation of Cu(II) adsorption onto poly(GDMA-co-MAA) nanospheres with the time. Conditions: $CuCl_2.2H_2O$: 200 mg, poly(GDMA-co-MAA) nanospheres: 100 mg, DI water: 40 mL. Stirring rate: 300 rpm, Room temperature.

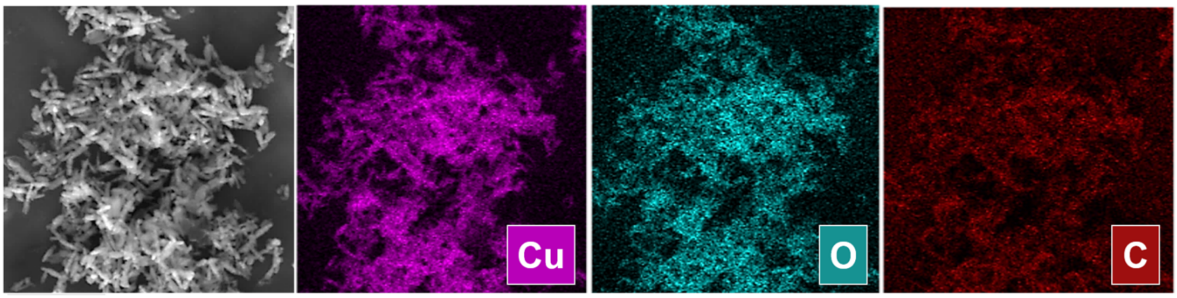

Figure S3. Energy-dispersive X-ray spectroscopy images of CuO NSs.

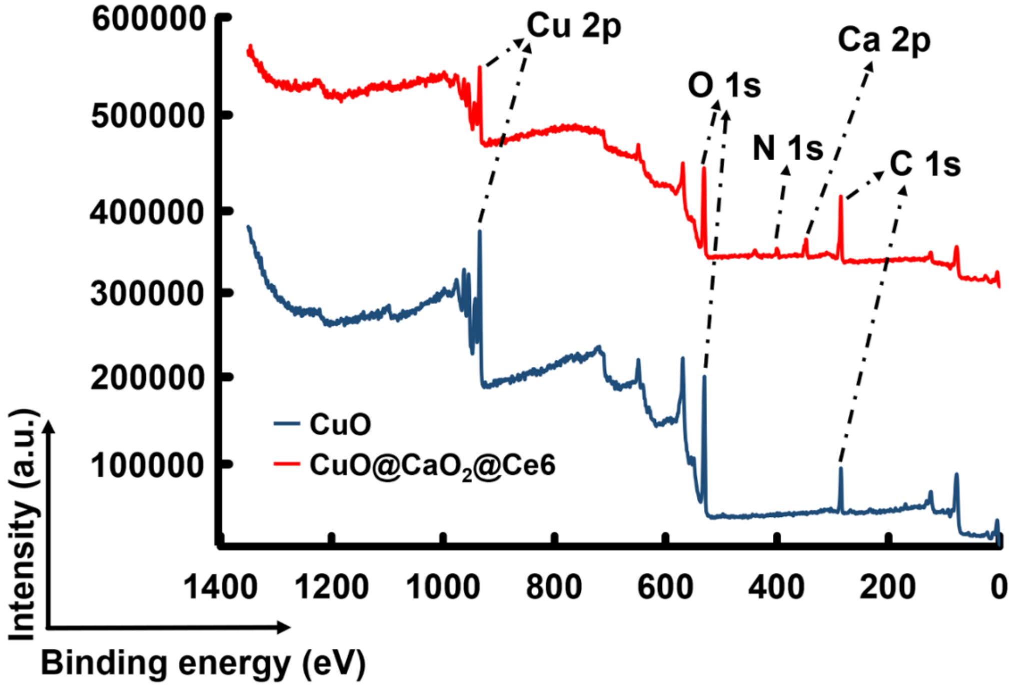

Figure S4. Survey X-ray photoelectron spectroscopy of CuO and CuO@CaO_2@Ce6 NSs.

Table S1. Surface atomic compositions of CuO and CuO@CaO_2@Ce6 NSs determined by XPS.

Surface atomic percentage (%)					
**Sample**	**Cu 2p3**	**Ca 2p3**	**C 1s**	**O 1s**	**N 1s**
CuO	16.37	-	32.57	51.06	-
CuO@CaO_2@Ce6	6.31	3.35	52.30	33.24	4.80

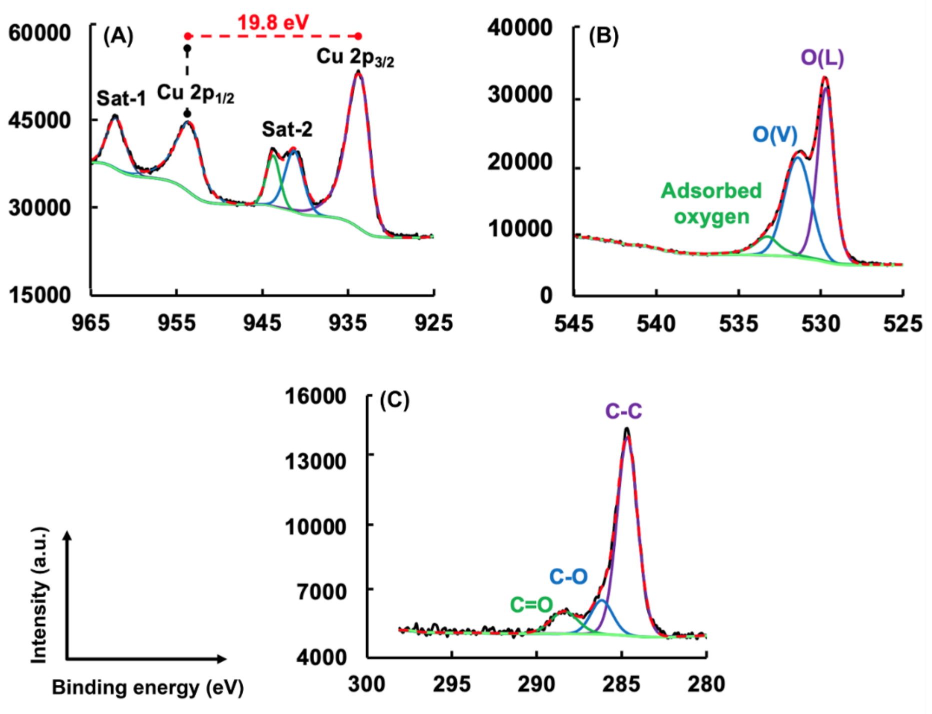

Figure S5. X-ray photoelectron spectroscopy of CuO NSs. (A) Cu 2p scan, (B) O 1s scan, (C) C 1s scan.

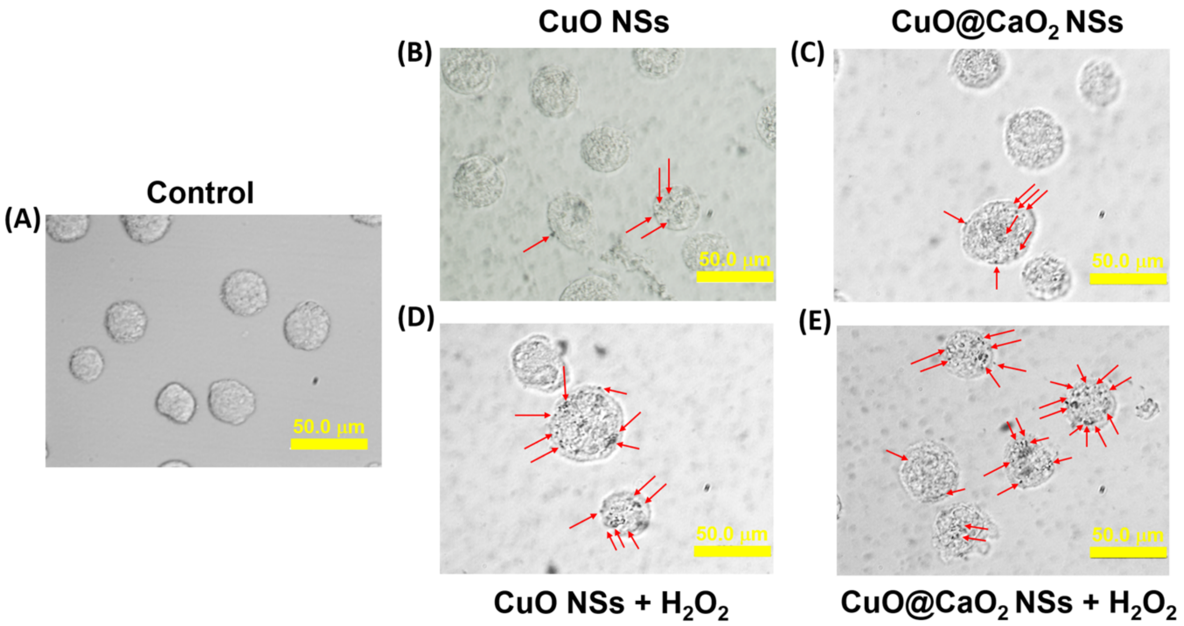

Figure S6. The effect of nanomotor function on the interaction of CuO and CuO@CaO_2 NSs with T98G cells. The inverted microscopy images of (A) non-adherent T98G cell suspension containing no NSs and no H_2O_2, (B) non-adherent T98G cell suspension containing only CuO NSs, (C) non-adherent T98G cell suspension containing only CuO@CaO_2 NSs, (D) non-adherent T98G cell suspension containing CuO NSs and 1 mM H_2O_2, and (E) non-adherent T98G cell suspension containing CuO@CaO_2 NSs and 1 mM H_2O_2. Cell density: $2x10^4$, Concentration of NSs: 0.5 mg/mL, Interaction time: 10 min, 37°C.

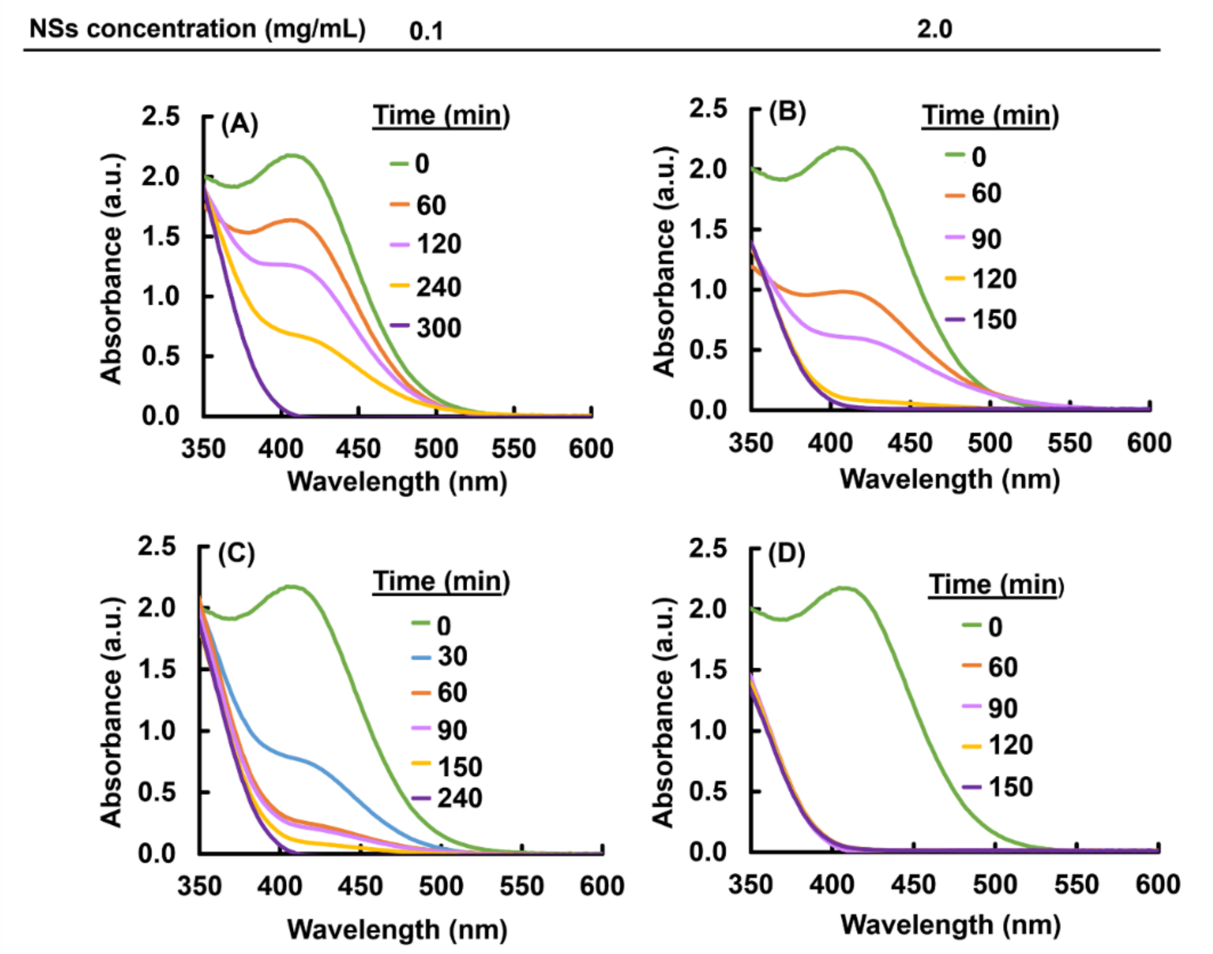

Figure S7. Sample UV-Vis spectra recorded for GSH depletion using DTNB as the reactive probe with low and high concentrations of CuO and CuO@CaO_2. (A) CuO NSs (0.1 mg/ mL), (B) CuO NSs (2.0 mg/ mL), (C) CuO@CaO_2 NSs (0.1 mg/ mL) and (D) CuO@CaO_2 NSs (2.0 mg/ mL).

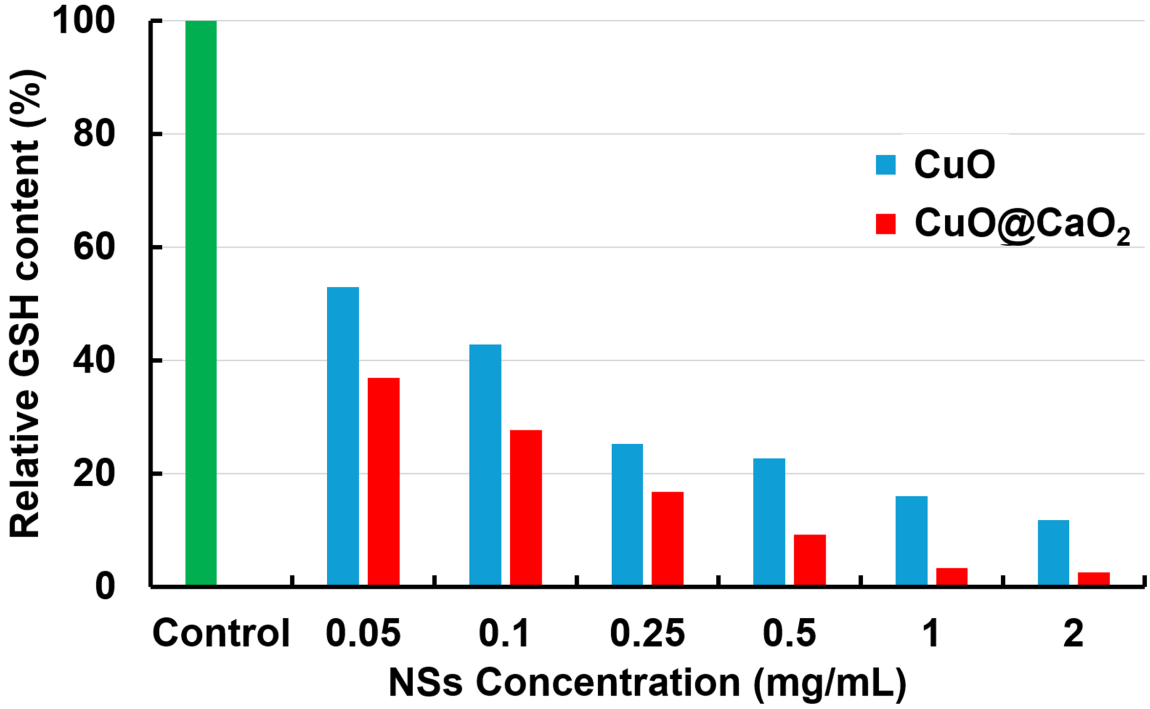

Figure S8. The variation of intracellular GSH content with the concentration of CuO and $CuO@CaO_2$ NSs. Conditions: Cell density: 4×10^4 cells/well, Time: 60 min.

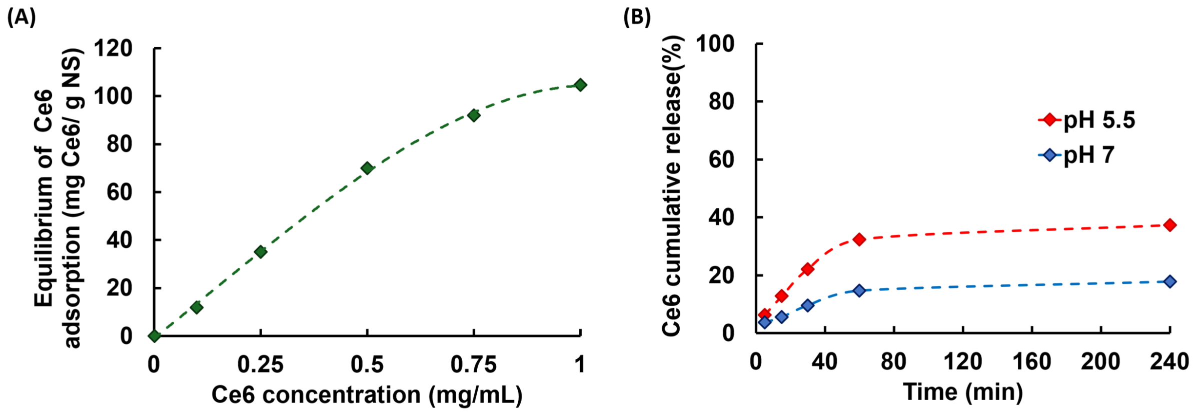

Figure S9. (A) The variation of equilibrium Ce6 adsorption onto $CuO@CaO_2$ NSs with the initial Ce6 concentration, (B) The cumulative release of Ce6 from $CuO@CaO_2@Ce6$ NSs in phosphate buffer at pH 5.5 and 7.0.

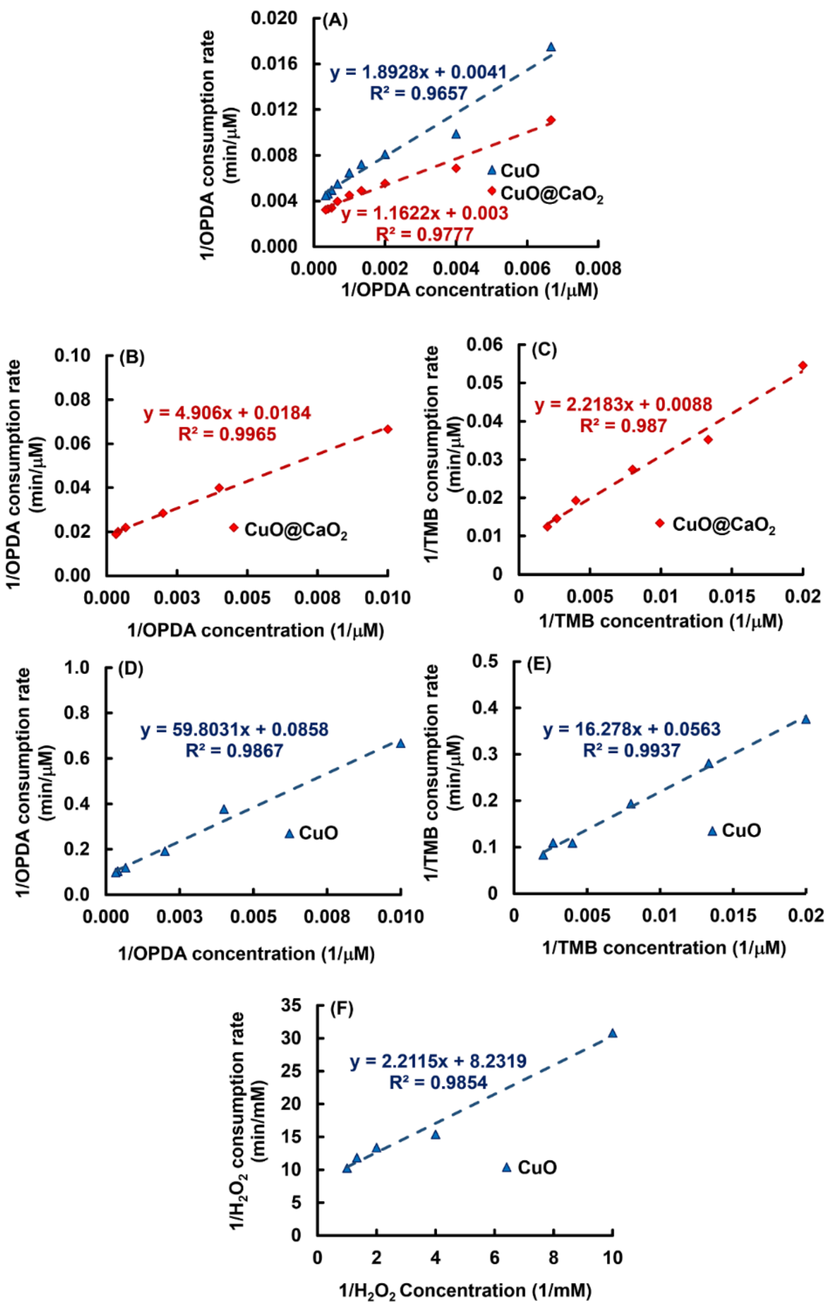

Figure S10. Lineweaver-Burk plots for (A) POD-like activities of CuO and CuO@CaO_2 NSs, (B) self POD-like activity of CuO@CaO_2 NSs without adding H_2O_2 using OPDA as the substrate at pH 7.0, (C) self- POD-like activity of CuO@CaO_2 NSs without adding H_2O_2 using TMB as the substrate at pH 5.0, (D) OD-like activity of CuO NSs using OPDA as the substrate at pH 7.0, (E) OD-like activity of CuO NSs using TMB as the substrate at pH 5.0, and (F) CAT-like activity of CuO NSs. Nanozyme concentration: 2.0 mg/mL. Temperature: 25°C.

Table S2. Comparison of K_m and V_{max} values of CuO and $CuO@CaO_2$ NSs

Catalyst	Substrate type	K_m (µM)	V_{max} (µM/min)
		**POD-like activity**	
CuO	OPDA	460.98	243.90
$CuO@CaO_2$	OPDA	386.68	333.33
		**Self POD-like activity**	
$CuO@CaO_2$	OPDA	266.63	54.35
$CuO@CaO_2$	TMB	252.07	113.64
		**OD-like activity**	
CuO	OPDA	11.66	697.01
CuO	TMB	17.76	289.13
		**CAT-like activity**	
CuO	H_2O_2	269	122

POD: Peroxidase, OD: Oxidase, CAT:Catalase

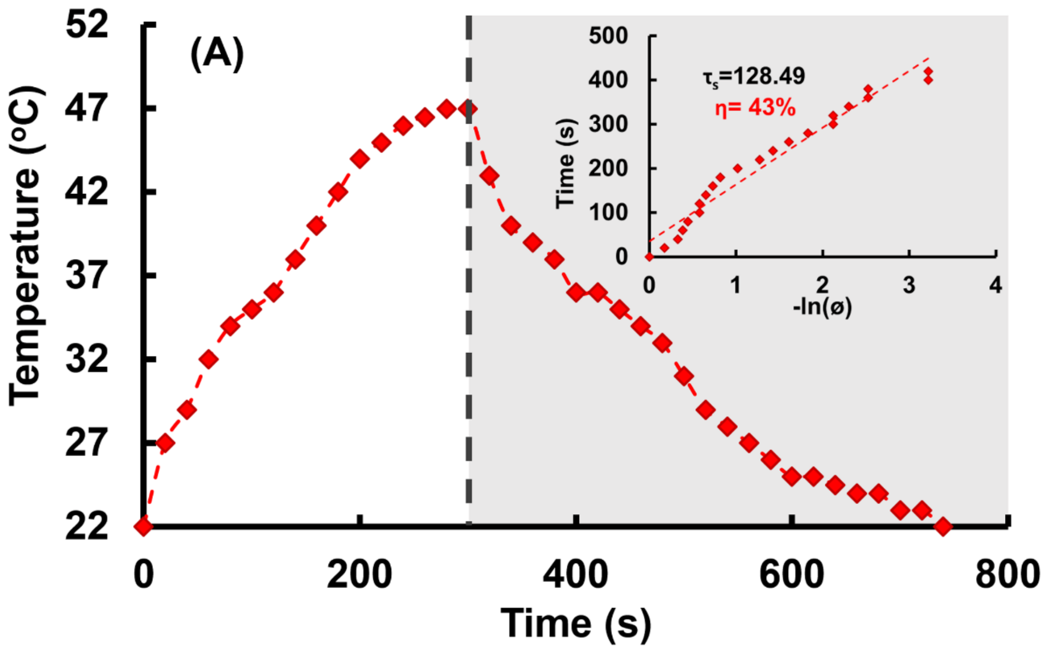

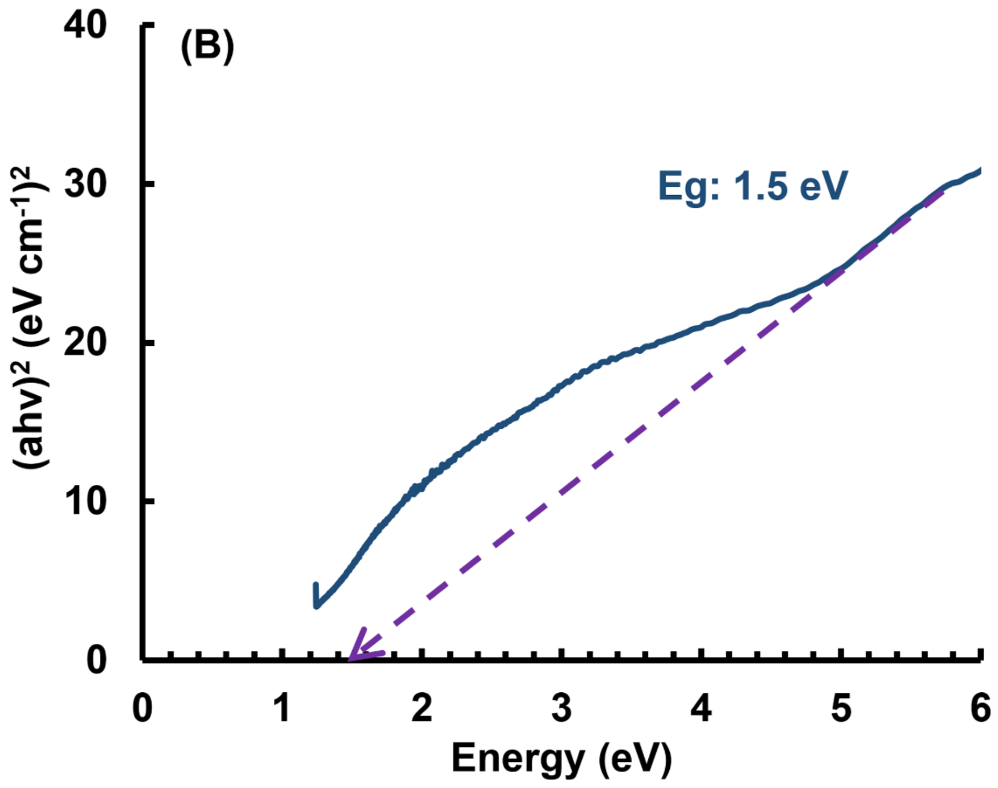

Figure S11. (A) A typical consecutive heating cooling cycle obtained using CuO@CaO_2@Ce6 NSs at a concentration of 1.0 mg/mL and the determination of photothermal efficiency (η) of CuO@CaO_2@Ce6 NSs according to the time constant method (Inset), (B) The Tauc plot sketched for CuO NSs for the determination of band gap energy.

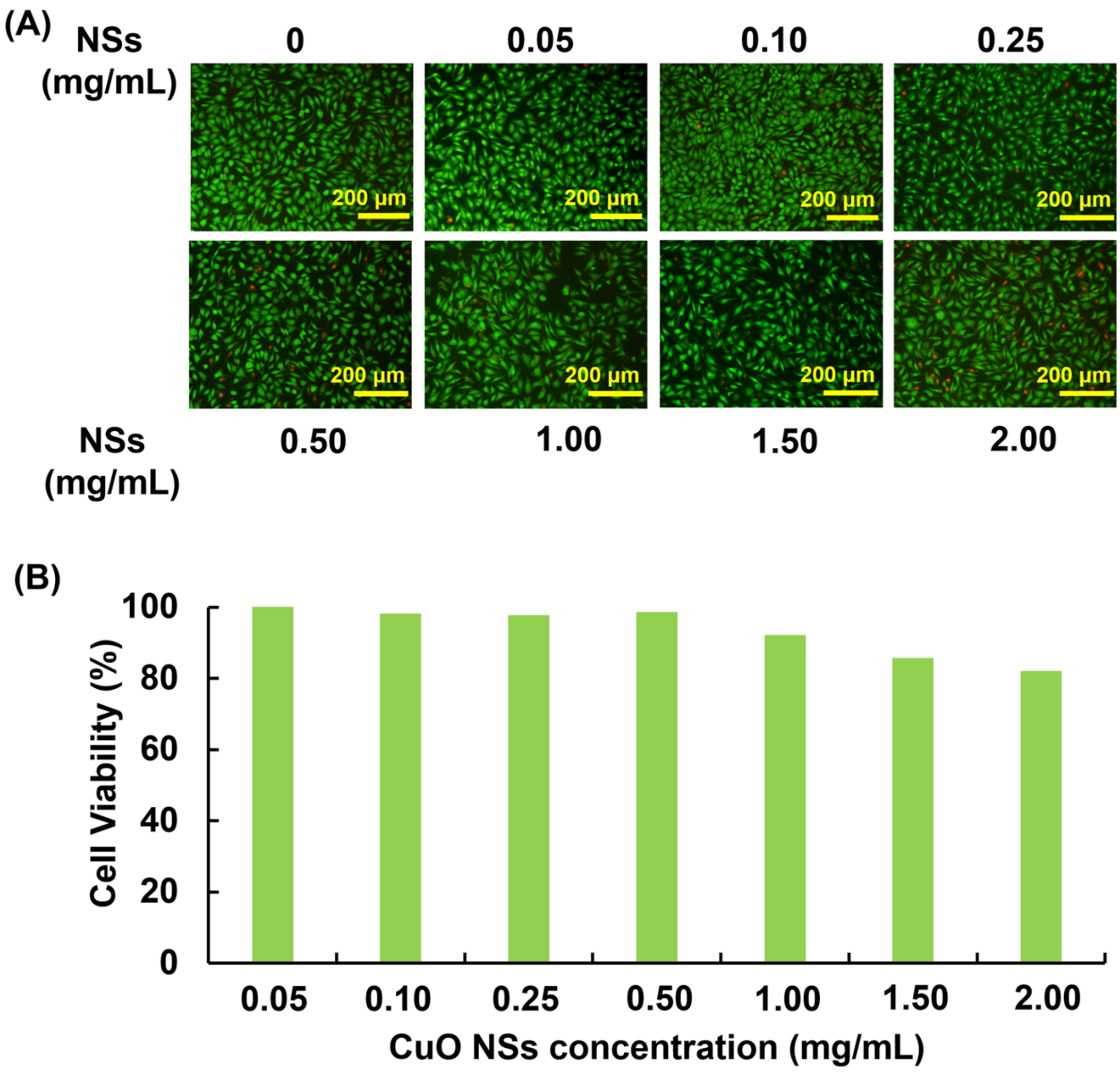

Figure S12. The viability of L929 cells with CuO NSs at different concentrations. (A) Live/dead L929 cell images taken after dual cell staining with AO/PI system, after treatment with CuO NSs with different concentrations. Scale bar: 200 μm, L929 cell density: $2x10^4$ cells/well. (B) MTT results exemplifying the viability of L929 cells after treatment with CuO NSs with different concentrations. The control image was obtained without including CuO NSs in the well.

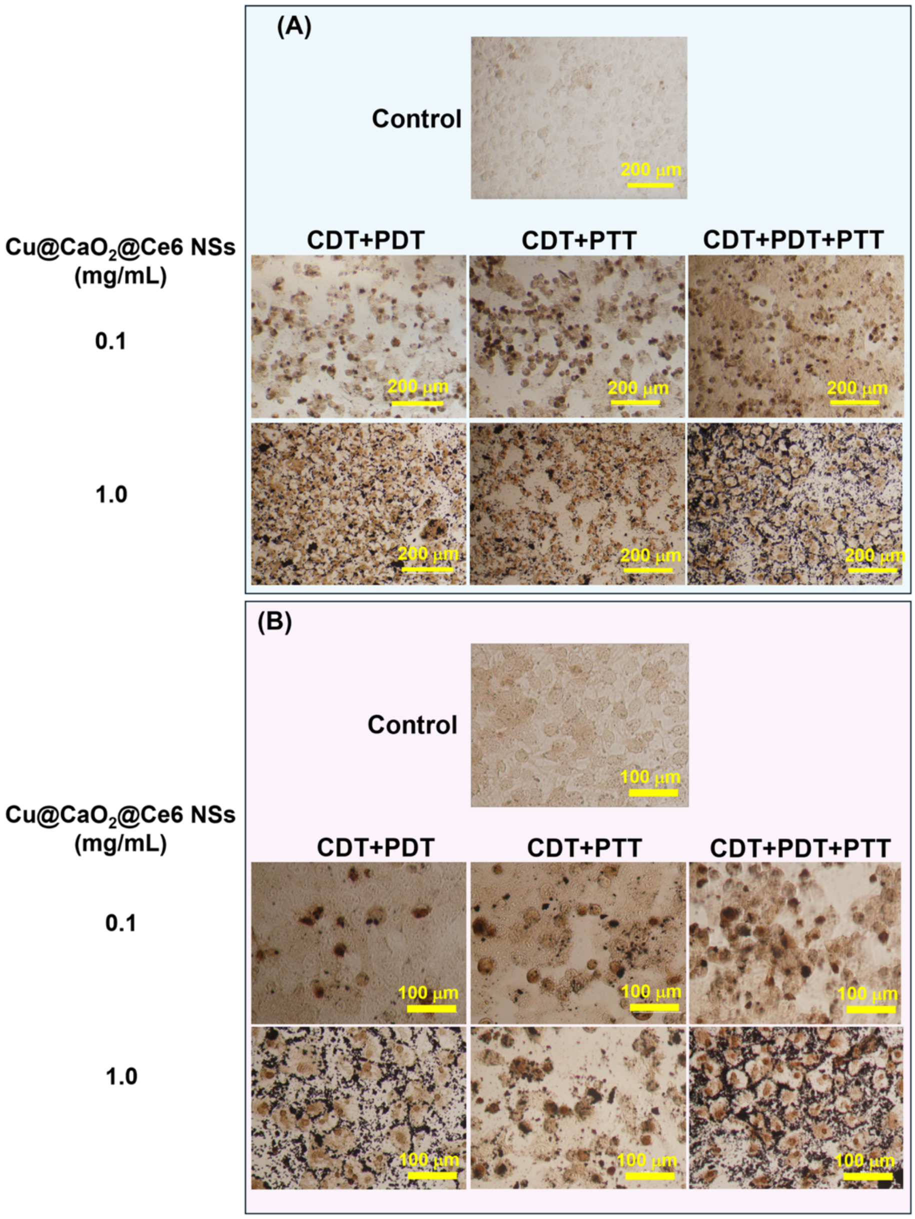

Figure S13. Representative inverted microscope images of apoptotic T98G cells identified by terminal deoxynucleotidyl Transferase-mediated dUTP Nick End Labeling (TUNEL) assay. Magnification: Panel A: X100, Panel B: X200. Conditions: T98G concentration: 2×10^4 cells/well, LED irradiation time: 7 min (0.8 W) for chemodynamic + photodynamic effects, NIR laser (808 nm) irradiation time: 5 min (1.3 W/cm^2) for chemodynamic + photothermal effects. LED irradition time: 7 min (0.8 W) + NIR laser (808 nm) irradiation time: 5 min (1.3 W/cm^2) for chemodynamic + photodynamic + photothermal effects. Concentration of CuO@CaO_2@Ce6 NSs: 0.1 and 1.0 mg/mL.

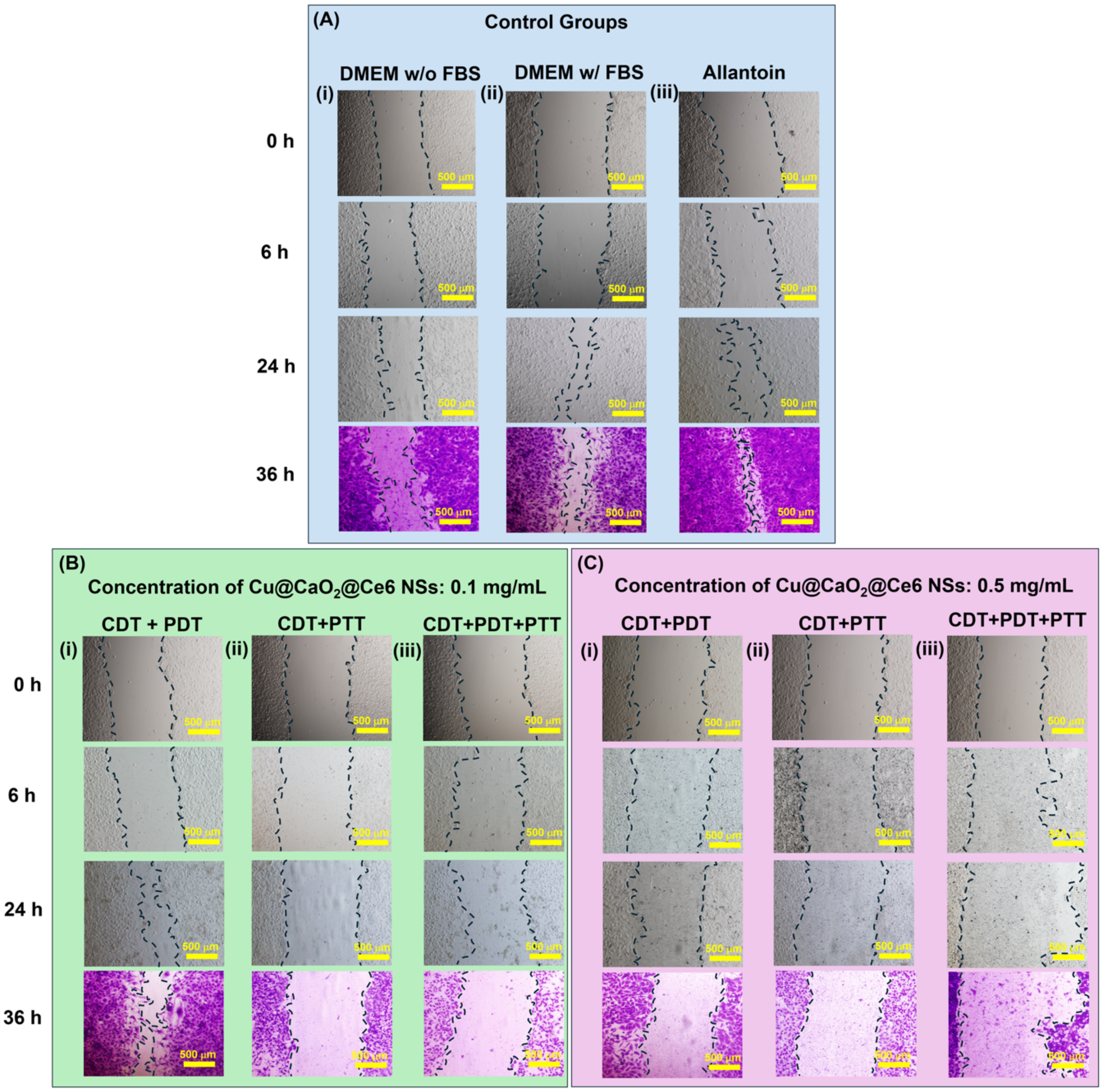

Figure S14. The inverted optical microcopy images of T98G cells subjected to scratch assay for cell proliferation and migration. Panel A: The images of control groups prepared with (i) DMEM without FBS, (ii) DMEM with FBS, (iii) DMEM containing allantoin, Panel B: The images of cells interacted with 0.1 mg/mL of CuO@CaO_2@Ce6 NSs by applying (i) chemodynamic + photodynamic, (ii) chemodynamic+ photothermal and (iii) chemodynamic + photodynamic + photothermal effects. Panel C: The images of cells interacted with 0.5 mg/mL of CuO@CaO_2@Ce6 NSs by applying (i) chemodynamic+ photodynamic, (ii) chemodynamic+ photothermal and (iii) chemodynamic + photodynamic + photothermal effects.
